# The complete chloroplast genome of *Onobrychis gaubae* (Fabaceae-Papilionoideae): comparative analysis with related IR-lacking clade species

**DOI:** 10.1186/s12870-022-03465-4

**Published:** 2022-02-19

**Authors:** Mahtab Moghaddam, Atsushi Ohta, Motoki Shimizu, Ryohei Terauchi, Shahrokh Kazempour-Osaloo

**Affiliations:** 1 Department of Plant Biology, Faculty of Biological Sciences, Tarbiat Modares University, 14115-154 Tehran, Iran; 2 Graduate School of Agriculture, Kyoto University, Kyoto, 617-0001 Japan; 3 Iwate Biotechnology Research Center, Kitakami, Iwate, 024-0003 Japan

**Keywords:** Hypervariable region, IRLC, *Onobrychis*, Phylogenetic relationship, Plastome

## Abstract

**Background:**

Plastome (Plastid genome) sequences provide valuable markers for surveying evolutionary relationships and population genetics of plant species. Papilionoideae (papilionoids) has different nucleotide and structural variations in plastomes, which makes it an ideal model for genome evolution studies. Therefore, by sequencing the complete chloroplast genome of *Onobrychis gaubae* in this study, the characteristics and evolutionary patterns of plastome variations in IR-loss clade were compared.

**Results:**

In the present study, the complete plastid genome of *O. gaubae*, endemic to Iran, was sequenced using Illumina paired-end sequencing and was compared with previously known genomes of the IRLC species of legumes. The *O. gaubae* plastid genome was 122,688 bp in length and included a large single-copy (LSC) region of 81,486 bp, a small single-copy (SSC) region of 13,805 bp and one copy of the inverted repeat (IR_b_) of 29,100 bp. The genome encoded 110 genes, including 76 protein-coding genes, 30 transfer RNA (tRNA) genes and four ribosome RNA (rRNA) genes and possessed 83 simple sequence repeats (SSRs) and 50 repeated structures with the highest proportion in the LSC. Comparative analysis of the chloroplast genomes across IRLC revealed three hotspot genes (*ycf*1, *ycf*2, *clp*P) which could be used as DNA barcode regions. Moreover, seven hypervariable regions [*trn*L(UAA)-*trn*T(UGU), *trn*T(GGU)-*trn*E(UUC), *ycf*1, *ycf*2, *ycf*4, *acc*D and *clp*P] were identified within *Onobrychis*, which could be used to distinguish the *Onobrychis* species. Phylogenetic analyses revealed that *O. gaubae* is closely related to *Hedysarum*. The complete *O. gaubae* genome is a valuable resource for investigating evolution of *Onobrychis* species and can be used to identify related species.

**Conclusions:**

Our results reveal that the plastomes of the IRLC are dynamic molecules and show multiple gene losses and inversions. The identified hypervariable regions could be used as molecular markers for resolving phylogenetic relationships and species identification and also provide new insights into plastome evolution across IRLC.

**Supplementary Information:**

The online version contains supplementary material available at 10.1186/s12870-022-03465-4.

## Background

Chloroplast is a vital organelle in plant cells that plays an important role in plant carbon fixation and numerous metabolic pathways [1, 2]. In angiosperms, the chloroplast genome (plastome) typically has a circular structure that ranges from 120 to 180 kb in length. Plastomes mostly exhibit a quadripartite structure in which a pair of inverted repeats (IRa and IRb; usually around 25 kb, but can vary from 7 to 88 kb each) separate the large single-copy (LSC, ca. 80 kb) and the small single-copy (SSC, ca. 20 kb) regions [1, 2]. Most plastomes encode 80 protein-coding genes primarily involved in photosynthesis and other biochemical processes along with 30 tRNA and 4 rRNA genes [3, 4]. In contrast to mitochondrial and nuclear genomes, the plastomes across seed plants are highly conserved with respect to gene content, structure and organization [5, 6]. However, mutations including duplications, rearrangements, and losses have been reported at the genome and gene levels among some angiosperm lineages, including Asteraceae [7], Campanulaceae [8], Onagraceae [9], Fabaceae [10] and Geraniaceae [11].

Fabaceae (legumes) is the third-largest family of angiosperms which shows much extensive structural variation in the plastid genome [12]. Currently accepted classification of the legumes based on plastid gene *mat*K includes six subfamilies: Caesalpinioideae, Cercidoideae, Detarioideae, Dialioideae, Duparquetioideae, and Papilionoideae [13]. Gene content and gene order among plastomes of subfamilies are highly conserved and similar to the ancestral angiosperm genome organization except for Papilionoideae, which exhibits numerous rearrangements and gene/intron losses and has smaller genomes [5]. In this subfamily, a loss of one of the IRs [14], the presence of many repetitive sequences [15] and the presence of a localized hypermutable region [15, 16] have been documented. The Papilionoideae is further divided into seven major clades [the *Cladrastis*, Genistoids, Dalbergioids, Mirbelioids, Millettioids, Robinioids and the inverted-repeat lacking clade (IRLC)] and several tribes [14]. IRLC is the largest legume lineage which contains over 4000 species in 52 genera and nine tribes [14, 17–20]. Species within the IRLC reveal multiple gene/intron losses [15, 21], several sequence inversions [10], gene transfer to the nucleus [15, 22] and localized hypermutation [15, 16]. The presence of genomic rearrangements along with nucleotide and structural variations in the IRLC plastomes have made it an excellent plant model for genome evolution studies.

Recently, with the advent of next-generation sequencing (NGS) technology, plastomes of several taxa from different tribes in this clade have been sequenced. The majority of IRLC plastomes sequenced to date were restricted to agricultural/medicinal species (from the tribes Fabeae, Trifolieae, Caraganeae and Galegeae) or the plant model *Medicago truncatula *[23]. Thus, it is essential to investigate the members from other lineages to better understand plastome evolution within the IRLC, and more broadly within Papilionoideae. The plastid genome of the tribe Hedysareae has not been considered in previous studies. Members of Hedysareae are commonly restricted to Eurasia, N America, and the Horn of Africa with Socotra and are widely used as forage plants due to their high protein content [24–26]. In the tribe Hedysareae [24] with nine genera, the plastomes of some *Hedysarum* species and only one species of *Onobrychis* (*O*. *viciifolia* within subgenus *Onobrychis*) have been reported. *Onobrychis* is the second largest genus after *Hedysarum* in the tribe Hedysareae. *Onobrychis* is composed of two subgenera [*Onobrychis* and *Sisyrosema* (Bunge ex Boiss.) Sirj.] and has more than 130 species [25]. This genus mainly distributed throughout temperate and subtropical regions of Eurasia, N and NE Africa [26]. In the present study, the complete plastome of *Onobrychis gaubae* Bornm. was sequenced (GenBank accession number: LC647182). *O*. *gaubae* belongs to the subgenus *Sisyrosema* and is a polymorphic species restricted to the southern slopes of the Alborz mountain range, Iran [25, 27]. The main goal of this study is to assemble the chloroplast genome of *O. gaubae*, and to annotate the genome and characterize its structure to provide a new genomic resource for this species. We also performed comparative analyses of the genome and phylogenetic reconstruction to evaluate the sequence divergence in the plastomes across the IR-lacking clade.

## Results

### Characteristics of the chloroplast genome of *O. gaubae*

**Fig. 1:**
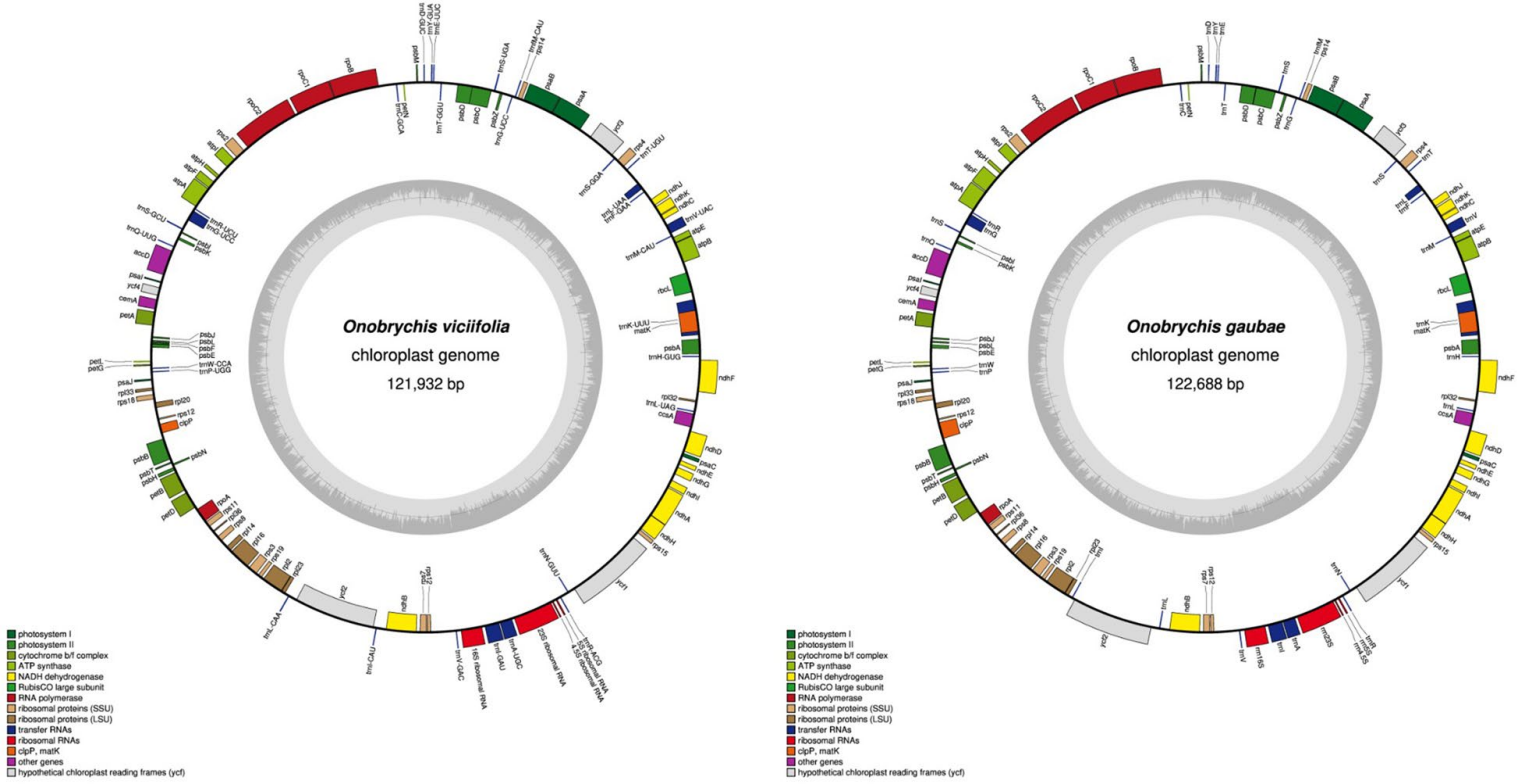
Gene map of two *Onobrychis* species chloroplast genome [*O*. *viciifolia* (MW007721) [29] and *O*. *gaubae* (LC647182)]. The genes drawn outside and inside of the circle are transcribed in clockwise and counterclockwise directions, respectively. Genes were colored based on their functional groups. The inner circle shows the structure of the chloroplast: The large single copy (LSC), small single copy (SSC) and inverted repeat (IR) regions. The gray ring marks the GC content with the inner circle marking a 50% threshold. Asterisks mark genes that have introns

The number of paired-end raw reads obtained by the Illumina HiSeq 2000 system is 43,189,861 for *O*. *gaubae* sample. The plastid genome of *O. gaubae* with 122,688 bp in length and having only one copy of the IR region is similar to those of other IRLC species. In this context, the lack of *rps*16 and *rpl*22 genes and intron 1 of *clp*P in the plastome of *O*. *gaubae* are noted; these genes, are absent from the chloroplast genomes of entire IRLC[21, 22, 28]. The assembled chloroplast genome of *O*. *gaubae* contained 110 genes, including 76 protein-coding genes, 30 transfer RNA (tRNA) genes and four ribosome RNA (rRNA) genes (Fig. 1, Table 1). The LSC (79,783 bp), SSC (13,805 bp) and IR (29,100 bp) regions along with the locations of 110 genes in the chloroplast genome are shown in Fig. 1.

**Table 1:** Genes predicted in the chloroplast genome of *O*. *gaubae*

Category of genes	Group of genes	Name of genes
Self-replication	Large subunit of ribosomal proteins	*rpl*14, *rpl*16*, *rpl*2*, *rpl*20, *rpl*23, *rpl*32, *rpl*33, *rpl*36
	Small subunit of ribosomal proteins	*rps*2, *rps*3, *rps*4, *rps*7, *rps*8, *rps*11, *rps*12*, *rps*14, *rps*15, *rps*18, *rps*19
	DNA-dependent RNA polymerase	*rpo*A, *rpo*B, *rpo*C1*, *rpo*C2
	Ribosomal RNA genes	*rrn*16S, *rrn*23S, *rrn* 4.5S, *rrn* 5S
	Transfer RNA genes	30 *trn* genes (5 contain an intron)
Genes for photosynthesis	Subunits of NADH-dehydrogenase	*ndh*A*, *ndh*B*, *ndh*C, *ndh*D, *ndh*E, *ndh*F, *ndh*G, *ndh*H, *ndh*I, *ndh*J, *ndh*K
	Subunits of photosystem I	*psa*A, *psa*B, *psa*C, *psa*I, *psa*J
	Subunits of photosystem II	*psb*A, *psb*B, *psb*C, *psb*D, *psb*E, *psb*F, *psb*H, *psb*I, *psb*J, *psb*K, *psb*L, *psb*M, *psb*N, *psb*T, *psb*Z
	Subunits of cytochrome b/f complex	*pet*A, *pet*B*, *pet*D*, *pet*G, *pet*L, *pet*N
	Subunits of ATP synthase	*atp*A, *atp*B, *atp*E, *atp*F*, *atp*H, *atp*I
	Subunit of rubisco	*rbc*L
Other genes	Maturase K	*mat*K
	Envelope membrane protein	*cem*A
	Subunit of Acetyl-CoA-carboxylase	*acc*D
	C-type cytochrome synthesis gene	*ccs*A
	Protease	*clp*P*
Genes of unkown function	Conserved hypothetical chloroplast open reading frames	*ycf*1, *ycf*2, *ycf*4, *ycf*3**

The number of asterisks after the gene names indicates the number of introns contained in the genes

A total of 16 genes (each separately) in *O*. *gaubae* chloroplast genome have only one intron, whereas *ycf*3 exhibits two introns (Additional File 1: Table S1). *rps*12 gene is a trans-splicing gene which does not have introns in the 3’-end. The *trn*K-UUU has the largest intron encompassing the *mat*K gene, with 2,495 bp, whereas the intron of *trn*L-UAA is the smallest intron (542 bp). The *O*. *viciifolia* plastome with 121,932 bp in length is very similar in gene contents, order and orientation to *O*. *gaubae*. The chloroplast genome of *O*. *viciifolia* has two major structural differences from *O*. *gaubae*: lack of the *atp*F intron and inversion of *ycf*2/*trn*I(CAU)/*trn*L(CAA) genes.

The length of plastome in the IRLC taxa in this study ranged from 121,020 to 130,561 bp. All plastomes exhibited the typical structure of IR-loss clade composed of LSC region (79,916 to 87,193), SSC region (13,383 to 14,187) and only one inverted repeat region (27,604 to 30,487) (Table 2).

**Table 2:** Chloroplast genome information from sampled IRLC species and the newly assembled *O. gaubae*

Species	Size (bp)	LSC (bp)	GC (%) (LSC)	SSC (bp)	GC (%) (SSC)	IR (bp)	GC (%) (IR)	GC (%) Total
*Astragalus mongholicus*	123,582	80,986	33.4%	13,773	29.9%	28,823	38.1%	34.1%
*Caragana microphylla*	130,029	85,436	33.3%	14,106	30.4%	30,487	38.8%	34.3%
*Carmichaelia australis*	122,805	80,588	33.5%	14,074	30.2%	28,143	38.6%	34.3%
*Cicer arietinum*	125,319	82,583	33%	13,820	29.9%	28,916	38.3%	33.9%
*Galega officinalis*	125,086	82,915	33.2%	13,347	30.5%	28,824	38.7%	34.2%
*Glycyrrhiza glabra*	127,943	84,714	33.1%	14,187	30.1%	29,042	39.6%	34.2%
*Hedysarum semenovii*	123,407	80,288	34.1%	13,679	30.5%	29,440	38.9%	34.9%
*Lens culinaris*	122,967	81,659	33.7%	13,833	30.2%	27,604	38.7%	34.4%
*Lessertia frutescens*	122,700	80,698	33.4%	13,750	29.9%	28,252	38.4%	34.2%
*Medicago sativa*	125,330	83,756	32.9%	13,383	30.2%	28,191	38.6%	34%
*Melilotus albus*	127,205	84,279	32.7%	13,806	29.8%	29,120	38.1%	33.6%
*Meristotropis xanthioides*	127,735	84,629	33.1%	14,150	30.1%	28,956	39.6%	34.2%
*Onobrychis gaubae*	122,688	79,783	33.8%	13,805	30.5%	29,100	38.8%	34.6%
*Onobrychis viciifolia*	121,932	78,986	33.8%	13,821	30.4%	29,125	38.8%	34.6%
*Oxytropis bicolor*	122,461	80,170	33.5%	14,017	30%	28,274	38.3%	34.2%
*Tibetia liangshanensis*	123,372	79,916	33.9%	13,513	30.6%	29,943	38.6%	34.7%
*Wisteria floribunda*	130,561	87,193	33.2%	14,127	30%	29,628	39.4%	34.4%

*LSC* Large Single Copy, *SSC* Small Single Copy, *IR* Inverted Repeat

Gene order and gene/intron content in plastomes of all the IRLC taxa are highly conserved. The overall GC content of the *O*. *gaubae* chloroplast genome sequence was 34.6%, which is consistent with other IRLC species, whose plastomes have GC-contents ranging from 33.6% to 35.1% (Table 2). Different GC content occurs in the LSC (32.7%—34.1%), SSC (29.8%—30.6%) and IR (38.1%—39.6%) regions (Table 2).

Sequencing, assembly and annotation confirm that the complete plastome of *O*. *gaubae* lacks the IRa region. Lack of this region is confirmed by PCR and Sanger sequencing for *O*. *gaubae*. PCR amplification is expected from the primers located in the *ndh*F-*psb*A region (IR/SSC boundary) and *rps*19-*rpl*2 region (LSC/IR boundary) for the species without IRa copy. In the present study, Sanger sequenced PCR amplicons agree with the absence of IRa copy in the plastid genome of *O*. *gaubae*.

### Codon usage bias

The total coding DNA sequences (CDSs) were 81,121 bp in length and encoded 75 genes including 24,765 codons which belonged to 61 codon types. Codon usage was calculated for the protein-coding genes present in the *O*. *gaubae* cp genome. Phenylalanine was the most abundant amino acid, whereas Alanine showed the least abundance in this species (Additional File 1: Table S2). Most protein-coding genes employ the standard ATG as the initiator codon. Among the *O*. *gaubae* protein-coding genes, three genes used alternative start codons; ACG for *psb*L and *ndh*D, and GTG for *rps*8. A similar codon usage pattern was exhibited in *O*. *viciifolia* (Additional File 1: Table S3).

The chloroplast genomes of the IRLC were analyzed for their codon usage frequency according to sequences of protein-coding genes and relative synonymous codon usage (RSCU). RSCU is an important indicator to measure codon usage bias in coding regions. This value is the ratio between the actual observed values of the codon and the theoretical expectations. A codon with an RSCU value higher than 1.0 has a positive codon usage bias, while a value lower than 1.0 has a negative codon usage bias. When the RSCU value is equal to 1.0 it means that this codon is chosen equally and had no bias [30, 31]. The total number of codons among protein-coding genes in the IRLC species varies from 20,381 in *Hedysarum taipeicum* (as the smallest number) to 24,765 in *O. gaubae*. The most often used synonymous codon was AUU, encoding isoleucine, and the least used was CGC/CGG, encoding arginine (Additional File 2: Table S4). In the IRLC, the standard AUG codon was usually the start codon for the majority of protein-coding genes and UAA was the most frequent stop codon among three stop codons. Methionine (AUG) and tryptophan (UGG) showed RSCU = 1, indicating no codon bias for these two amino acids. The highest RSCU value was for UUA (~ 2.04) in leucine and the lowest was GGC (~ 0.35) in glycine. Leucine preferred six codon types (UUA, UUG, CUU, CUC, CUA, and CUG) and actually showed A or T (U) bias in all synonymous codons (Additional File 2: Table S4). The result of distributions of codon usage in the IRLC species showed that RSCU > 1 was recorded for most codons that ended with an A or a U, except for UUG codon, resulting in the bias for A/T bases. As well as, more codons with the RSCU value less than one, ended with base C or G. So, there is high A/U preference in the third codon of the IR-loss clade coding regions, which is a common phenomenon in cp genomes of vascular plants [32].

### Analysis of repeats

**Fig. 2:**
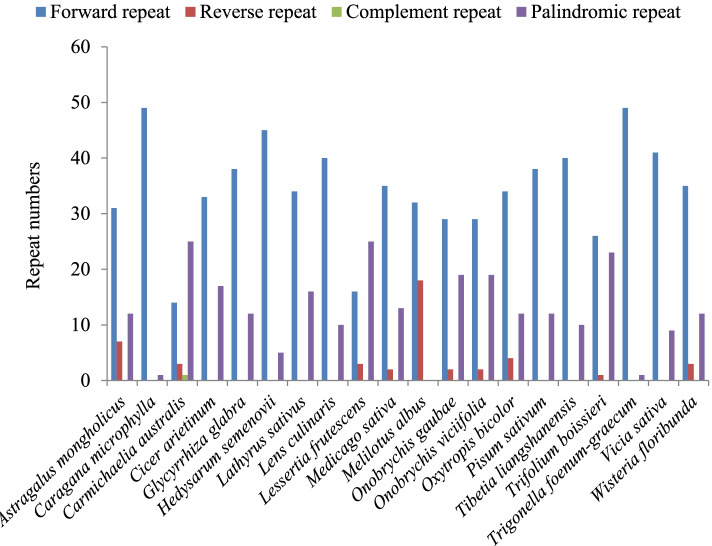
Analysis of repeated sequences in the IRLC species chloroplast genomes

Repeat analysis of *O*. *gaubae* plastome identified 50 repeat structures with lengths ranging from 30 to 179 bp. These structures included 29 forward repeats with lengths in the range of 30–179 bp, 19 palindromic repeats of 30–81 bp and two reverse repeats with a length of 31 and 37 bp (Additional File 3: Table S5). Among the 50 repeats, 66% are located in the LSC region, 18% in the IR region and 16% in the SSC region. Also, most of the repeats (42%) were found in coding regions (*acc*D, *psa*A, *psa*B, *psb*C, *psb*J, *ycf*1, *ycf*2, *ycf*4, *rps*12, *trn*R-UCU, *trn*K-UUU), 40% were distributed in the intergenic spacer regions (IGS) and 18% were located in the introns (*ndh*A, *rpl*16, *rps*12, *pet*B, *ycf*3). The pattern of repeat structures (both in frequency and location) in *O*. *gaubae* is similar to that of *O*. *viciifolia* (Additional File 3: Table S6). In the majority of the studied IRLC species, the most frequently observed repeats were forward, then palindromic, and the least reverse (Fig. 2).

In the IRLC species, the most abundant dispersed repeats identified were forward with lengths ranging from 30 to 50 bp. The longest repeats were also of the forward type, with the length of 560 bp were detected in the *Hedysarum taipeicum*, followed by *Vicia sativa* of 517 bp and *Caragana microphylla* of 455 bp, which were much longer than other species studied.

**Fig. 3:**
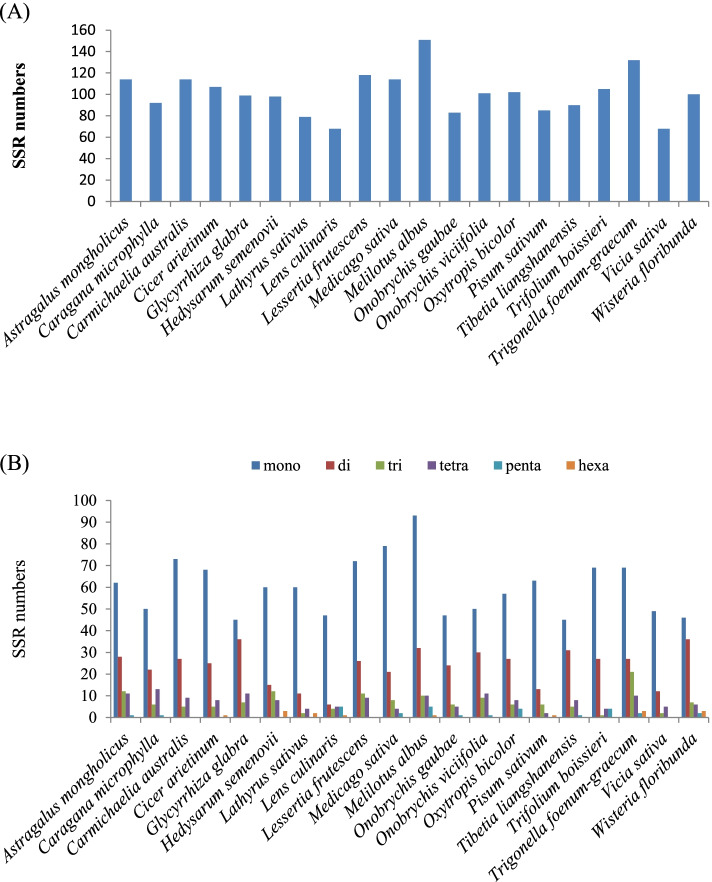
Analysis of perfect simple sequence repeats (SSRs) in the IRLC chloroplast genomes. **A** The number of SSRs detected in the IRLC chloroplast genomes; (**B**) The number of SSR types detected in the IRLC chloroplast genomes

Simple sequence repeats (SSRs), or microsatellites, are a type of tandem repeat sequences that contain 1–6 nucleotide repeat units and have wide distribution throughout the genome [31, 33]. Accordingly, microsatellites play a crucial role in the genome recombination and rearrangement. These nucleotide motifs show a high level of polymorphism that can be widely used for phylogenetic analysis, population genetics and species authentication [31, 34–36]. A total of 83 SSRs were detected in the *O*. *gaubae* plastome, which were composed by a length of at least 10 bp. Among them, 47 (56.62%) were mono-repeats, 24 (28.91%) were di-repeats, 6 (7.22%) were tri-repeats, five (6.02%) were tetra-repeats and one were penta-repeats (1.2%). No hexanucleotide SSRs was found in *O. gaubae* genome (Additional File 3: Table S7). *Onobrychis viciifolia* with 101 SSRs including 50 mono-repeats (49.5%), 30 (29.7%) di-repeats, nine (8.91%) tri-repeats, 11 (10.89%) tetra-repeats and one penta-repeat (0.99%), exhibited similar SSR distribution pattern in the plastome (Additional File 3: Table S8). The number of SSRs in the IRLC cp genomes (cpSSRs) ranged from 68 (*Vicia sativa* and *Lens culinaris*) to 151 (*Melilotus albus*) across the IRLC species (Fig. 3A). The mononucleotide repeats (P1) were identified at a much higher frequency, which varied from 45 (*Tibetia liangshanensis*, *Glycyrrhiza glabra*) to 93 (*Melilotus albus*) (Fig. 3B).

In the mononucleotide repeats, A/T motifs were the most abundant but no G/C motif was detected in the cp genome. Likewise, the majority of the dinucleotides and trinucleotides were found to be particularly rich in AT sequences.

### Sequence divergence analysis

**Fig. 4:**
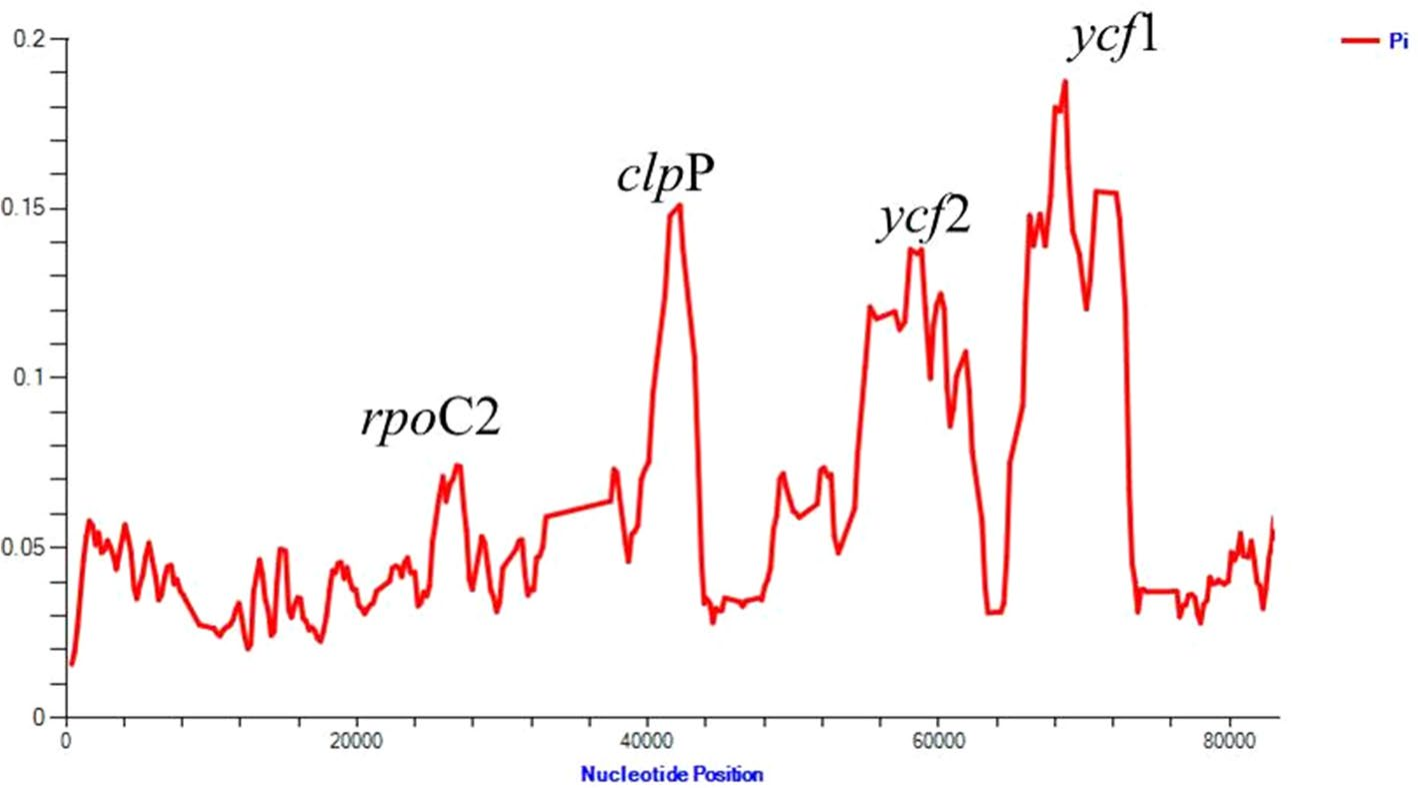
Nucleotide variability (%) values among the IRLC species (using for coding regions). Window length: 800 bp; step size: 200 bp. X-axis: Position of the midpoint of a window. Y-axis: Nucleotide diversity of each window

The average nucleotide diversity (Pi) among the protein-coding genes of 23 species of the IRLC was estimated to be 0.05736. Furthermore, comparison of nucleotide diversity in the LSC, SSC and IR regions indicated that the IR region exhibits the highest nucleotide diversity (0.11549) and the SSC region shows the least (0.04132). We detected three hyper-variable regions with *Pi* values > 0.1 among the IRLC species; *ycf*1 and *ycf*2 from IR region and *clp*P from LSC region (Fig. 4).

Among these, *ycf*1 encoding a protein of 1800 amino acids has the highest nucleotide diversity (0.18745). The average nucleotide diversity was also investigated between two *Onobrychis* plastid genome sequences. The average value of Pi between the *Onobrychis* species was estimated to be 0.05632 (Additional File 4: Fig. S1). High nucleotide variations were observed for the protein-coding regions *ycf*1, *ycf*2, *clp*P, *acc*D and *ycf*4 and intergenic regions such as *trn*L(UAA)-*trn*T(UGU) and *trn*T(GGU)-*trn*E(UUC). Sliding window analysis results revealed the same variable regions in the cp genome of the two *Onobrychis* species.

Moreover, mVISTA was used to compare whole chloroplast genome sequences of the IRLC species. We found that, similar to other plant species, the gene coding regions were more conserved than the noncoding regions (Additional File 5: Fig. S2). High nucleotide variations were observed across the IRLC for the protein-coding regions *ycf*1, *ycf*2 and *clp*P. Similar results were also obtained from the calculation of nucleotide diversity (Pi).

### Selection pressure analysis

In this study, the non-synonymous (Ka) to synonymous (Ks) rate ratio (Ka/Ks) was estimated for 75 protein-coding genes across the 28 IRLC species by using DnaSP v.6.12 [37] (Additional File 6: Table S9). In general, the Ka/Ks values were lower than 0.5 for almost all genes. The *ycf*4 gene which is involved in regulating the assembly of the photosystem I complex had the highest nonsynonymous rate, 0.165691, while the *ycf*1 gene with unknown functions had the highest synonymous rate, 0.181067. The Ka/Ks ratio (denoted as *ω*) is widely used as an estimator of selective pressure for protein-coding genes. *ω* > 1 indicates that the gene is affected by positive selection, *ω* < 1 indicates purifying (negative) selection, and *ω* equal to 1 indicates neutral mutation [38]. In the present study, the Ka/Ks ratio was calculated to be 0 for *psb*L gene which encodes one of the subunits of photosystem II. The Ka/Ks ratio indicates purifying selection in 73 protein-coding genes. The highest Ka/Ks ratio which indicates positive selection was observed in *acc*D gene which encodes a subunit of the acetyl-CoA carboxylase (ACCase) enzyme.

### Prediction of RNA editing sites

RNA editing as a post-transcriptional modification process, mainly occurs in chloroplasts and mitochondrial genomes. In higher plants, some chloroplast RNA editing sites which provide a way to create transcript and protein diversity are conserved [31].

RNA editing sites of *O. gaubae* plastid genes were predicted using Prep-CP prediction tool (Additional File 7: Table S10). In total, 58 editing sites were present in 19 chloroplast protein-coding genes and all of the editing sites were C-to-U conversions (Additional File 7: Table S10). Among them, nine editing sites, the highest number, were found in the region encoding *ndh*B gene followed by seven editing sites in *pet*B. There were six editing sites detected each in *ndh*A and *rpo*B genes. *acc*D, *ndh*G and *pet*D had three editing sites, and *ndh*D and *ndh*F had two editing sites. Two editing sites were also found in *ccs*A, *mat*K and *rpo*C1 genes. The remaining seven genes had only one editing site. The results showed that *ndh* genes exhibited the most abundant editing sites which were nearly 39.6% of the total editing sites. Furthermore, we predicted 65 RNA editing sites out of 22 plastid genes in chloroplast genomes of *O*. *viciifolia*. In this species, the highest number of editing sites belongs to the *pet*B, *rpo*C1 and *ndh*B genes with 9, 8 and 7 sites, respectively (Additional File 7: Table S11).

### Phylogenetic analysis

**Fig. 5:**
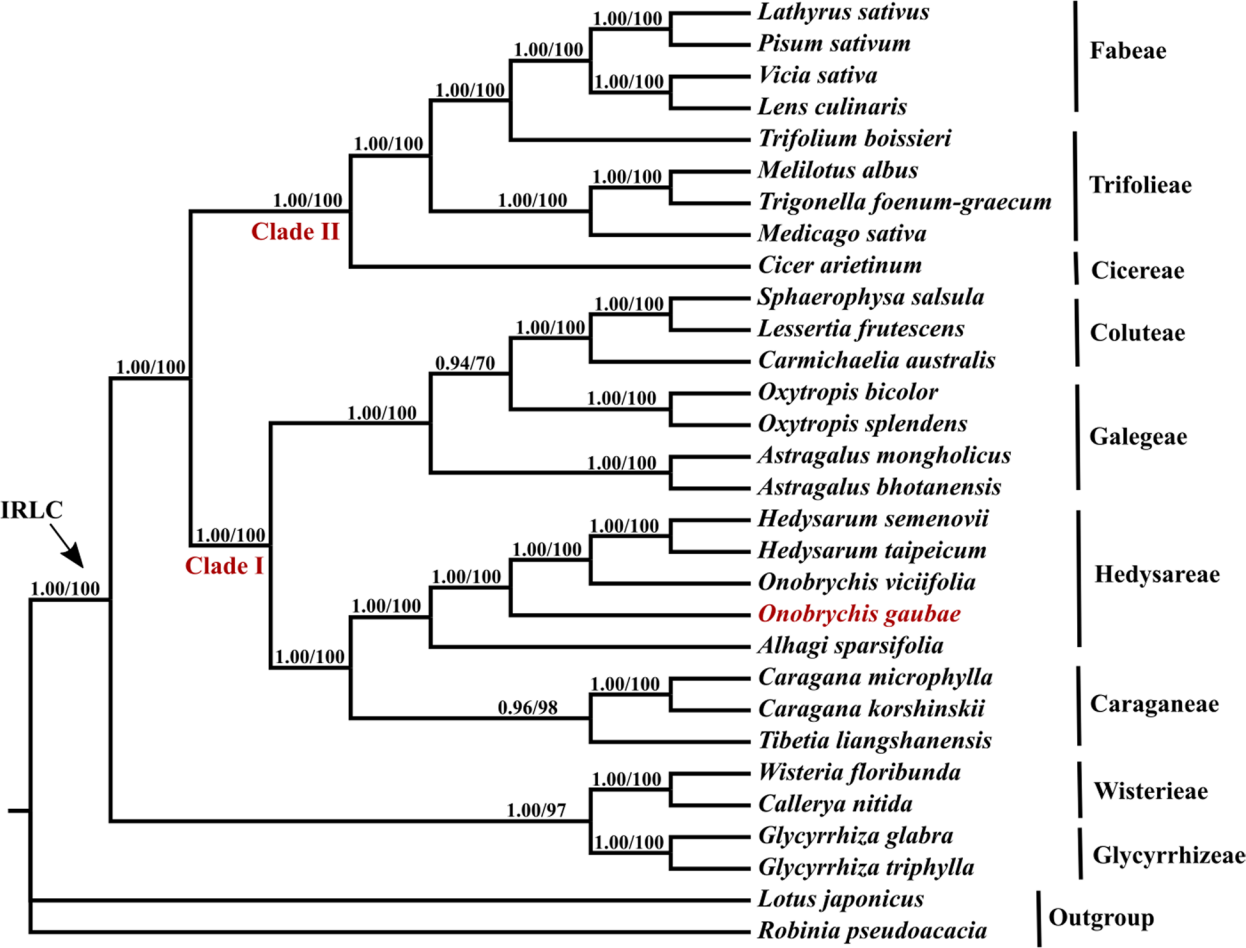
Fifty percent majority rule consensus tree resulting from Bayesian analysis of the 75 plastid genes of IRLC. The position of *Onobrychis gaubae* is shown in red. Numbers above branches are posterior probability and likelihood values, respectively

Phylogenetic relationships within the IRLC were reconstructed using the representative taxa (28 species from different tribes) and two species as outgroups based on 75 protein-coding genes of their chloroplast genomes. The total concatenated alignment length from the 75 protein-coding genes was 87,455 bp. The maximum likelihood (ML) analysis resulted in a well-resolved tree and the Bayesian inference yielded a well-resolved topology with high support values (Fig. 5).

The ML and Bayesian trees were largely congruent. The tribe Wisterieae [19] together with tribe Glycyrrhizeae [20], which formed a well-supported clade, were sister to the rest of the IRLC.

Following the basal group, the IRLC divided into two clades: clade I and II (Fig. 5). Clade I comprises tribes Caraganeae [17], Hedysareae [24] and Coluteae [18] as well as genera *Oxytropis* and *Astragalus*. Our results confirmed a close relationship among *O. gaubae*, *O. viciifolia* and *Hedysarum* species and showed *O. gaubae* phylogenetic position in the tribe Hedysareae. Furthermore, our plastid DNA analyses represent that *Oxytropis* is sister to the tribe Coluteae. Clade II contains tribes Cicereae, Trifolieae and Fabeae.

## Discussion

### General features of the *Onobrychis gaubae* plastid genome

In our study, we determined the first complete chloroplast genome sequence of *O*. *gaubae* within *O*. subgenus *Sisyrosema* using the Illumina platform and deposited in the GenBank (Fig. 1). Our assembly and annotation results showed that the length of the cp genome is 122,688 bp and its structure is similar to those of other IRLC species. Plastomes of *O*. *gaubae* and *O*. *viciifolia* are highly conserved and are similar with respect to genome organization and gene content. In this regard, one of the structural changes detected in the *O*. *viciifolia* cp genome is the loss of the *atp*F intron; whereas, *O*. *gaubae* possesses this intron. The *atp*F gene of *O*. *gaubae* is 1261 bp long with one intron of 702 bp, exon 1 of 144 bp and exon 2 of 415 bp. While the *atp*F gene of *O*. *viciifolia* is 558 bp long. Introns which are generally conserved regions among land plants play an important role in the expression of genes by increasing their transcription. Introns as the mobile genetic elements in the plastome, are mainly classified as either group I and group II. Group I and group II introns are derivatives of self-splicing RNA enzymes (ribozymes). Group I introns are present in rRNA, tRNA and protein-coding regions, while group II introns are found primarily in protein-coding genes [39, 40]. There are 17 to 20 introns classified under group II in the cp genome of land plants [40]. The *atp*F gene has a conserved group II intron which has been found in the most previously sequenced land plant plastomes [40]. The *atp*F intron is rarely lost in flowering plants but some intronless chloroplast genomes have been reported, including *Manihot* (Euphorbiaceae)[40], *Passiflora* (Passifloraceae) [41] and several taxa across the IRLC (*Colutea nepalensis*, *Lessertia frutescens*, *Oxytropis bicolor*, *O*. *racemosa* and *Sphaerophysa salsula*) [42]. The loss of intron in *atp*F gene is yet to be determined in other taxa of Papilionoideae and IR-lacking clade. It has been suggested that recombination between an edited mRNA and the *atp*F gene may be a possible mechanism for the loss of intron [40]. Structural variations such as intron presence/absence can be useful as a molecular marker to provide informative characters at low taxonomic levels in phylogenetic studies [22]. Another structural change in plastome of *O*. *viciifolia* is the inversion of *ycf*2/*trn*I(CAU)/*trn*L(CAA) genes. Among angiosperms, most of the plastid genome inversions are found in the LSC region [5], while plastome inversion in the *O*. *viciifolia* is located within the IR region. The same inversion has also occurred in the plastomes of two species of *Astragalus *[43]. Plastome inversions due to the relative rarity and easily determined homology (no homoplasy), are highly valuable and useful in phylogenetic studies [5]. The main cause of inversions is not fully understood, but intramolecular recombination between dispersed short inverted/direct repeats and tRNA genes is an accepted explanation [44, 45].

Numerous plastomes have now been sequenced that contains IRs with different sizes and other taxa that lack one copy of IR entirely such as the Inverted Repeat Lacking Clade (IRLC) in subfamily Papilionoideae of Fabaceae. As mentioned above, *Onobrychis* as a member of tribe Hedysareae, belongs to the IR-lacking clade. Plastome IRa loss in the IRLC taxa which is considered as a strong phylogenetic signal in the clade, has been confirmed several times in the previous studies [14, 46, 47]. In this study, to verify the lack of IRa region in *O*. *gaubae*, two primer pairs were designed. PCR amplification was only successful when using the diagnostic primers pair for the absence of the IRa.

Papilionoideae, in particular the IRLC, displays genomic structural variations which provide informative characters to increase phylogenetic resolution and make the taxon an excellent model for genome evolution studies [5, 22]. The plastomes of several members of the IRLC have regions with significant variations, rearrangements and accelerated mutation rates, including loss of introns from *rps*12 and *clp*P genes [21], absence of *rps*16 gene [28] and transfer/loss of *rpl*22 to the nucleus [21]. Numerous studies have also shown some other rearrangements in some IRLC taxa, such as loss of *acc*D gene in six species of *Trifolium *[10, 22], loss of *rpl*23 and *rpl*33 genes in some species of *Lathyrus*, *Pisum* and *Vicia *[34] and loss of *ycf*4 gene in some species of *Lathyrus* and *Pisum *[15, 16]. As revealed in other studies, there are several reasons for the occurrence of rearrangements in the plastome, such as the lack of one IR region, size variation of IR region and many tandemly repeated sequences [48]. For example, the loss of the *rps*16 gene was probably due to the presence of a nuclear *rps*16 copy, which contributed to the pseudogenization of the plastid copy [48]. Likewise, the lack or expansion of the *acc*D gene was explained by the presence of tandemly repeated sequences [6, 15].

As previously mentioned, the plastomes of Papilionoideae, particularly IR-loss clade, are not conserved in their genomic structure in terms of gene order and gene content and exhibit numerous rearrangements and gene/intron losses [5, 21, 22]. In this context, our results showed that the lengths of the IRLC plastid genomes ranged from 121,020 to 130,561 bp. This suggests that the IRLC cp genomes may have undergone different evolutionary processes such as gene/intron loss, insertion/deletion and IR/LSC/SSC expansion/contraction [49]. The plastomes among the IRLC taxa were similar in GC content but higher GC content was usually detected in the IR region compared to the other regions of cp genome, which is mainly due to the presence of rRNA genes (*rrn*23, *rrn*16, *rrn*5, *rrn*4.5) with high GC content (50%-56.4%) in IRs [6, 35]. One of the factors that shape codon usage biases in different organisms is the GC content in codon positions. Codon usage bias indicates the importance of molecular evolutionary phenomena. As mentioned above, codon usage patterns are similar between two *Onobrychis* species and also across the IRLC.

Whole plastid genome alignments can elucidate the level of sequence divergence and easily identify large indels, which are extremely useful for phylogenetic analyses and plant identification. In the present study, our results showed that the sequence divergence was distributed in the LSC and IR regions in the IRLC. Three highly variable regions (*clp*P, *ycf*1, *ycf*2) were observed with higher Pi values and were located in the LSC and IR regions, respectively. The gene *ycf*1 with the highest nucleotide diversity is more variable than *mat*K and it can be useful for molecular systematics at low taxonomic levels [50, 51]. Furthermore, several divergence hotspots between *Onobrychis* species were identified, including *ycf*1, *ycf*2, *clp*P, *acc*D, *ycf*4 (as the protein-coding regions) and *trn*L(UAA)-*trn*T(UGU) and *trn*T(GGU)-*trn*E(UUC) (as the intergenic regions). Several studies [14, 18, 52] analyzed the phylogenetic reconstructions of the IRLC species at various taxonomic levels based on different plastid genes such as *mat*K, *ndh*F and *rbc*L, the nuclear ribosomal ITS and the combined sequences of these genes/spacers. We could use the highly variable regions acquired from this study to develop the potential phylogenetic markers which can be useful for species authentication and reconstruction of phylogeny within different tribes/genera of the IR-lacking clade in further studies.

In this study, we found many repeat regions including forward repeats, palindromic repeats and reverse repeats, which could be important hotspots for genome reconfiguration. Forward types were the most frequent in the IR-loss clade. Furthermore, repeat sequences were mainly distributed in non-coding regions (IGS) across the IRLC. As mentioned above, repeat structures induce indels and substitutions resulting in the mutation hotspot in the reconfiguration of genome [6]; therefore, these repeats can provide valuable information for phylogenetic and population studies [31]. In the IR-loss clade, mononucleotide repeats were highly abundant and were mostly composed of A/T rather than G/C repeats. Strong A/T bias in SSR loci was also observed in other legumes such as *Vigna radiate *[53], *Arachis hypogaea *[54] and *Stryphnodendron adstringens *[35] which, like other plastomes of species, may contribute to the bias in base composition [6]. The results showed that SSR loci of LSC region appeared more frequently than SSC or IR regions, which may be hypothesized that this phenomenon is relevant to the lack of one IR region in the IR-loss clade. In general, cpSSRs show abundant variation and might provide useful information for detecting intra- and interspecific polymorphisms at the population level [33, 36].

### Plastid RNA editing prediction and Ka/Ks ratio

RNA editing is one of the post-transcriptional mechanisms which converts cytidine (C) to uridine (U) or U to C at specific sites of RNA molecules and modifies the genetic information from the genome in the plastids and mitochondria of land plants. RNA editing serves as a mechanism to correct missense mutations of genes by inserting, deleting and modifying nucleotides in a transcript [55]. In the present study, the editing sites were mostly observed in *ndh* genes. In this regard, the highest number of plastid editing sites was found in the *ndh* group genes in flowering plants [55]. Moreover, the *ndh* genes encoding a thylakoid Ndh complex, have been lost or pseudogenized in different species of algae, bryophytes, pteridophytes, gymnosperms, monocots, eudicots, magnoliids, and protists [56–58]. The RNA editing is probably important for the NDH protein complex function and may also lead to improved photosynthesis and display positive selection during evolution [55].

Moreover, we estimated the Ka/Ks for each gene in DnaSP v.6.12 [37]. Acceleration of the evolutionary rate was observed only in the *acc*D gene. Some previous studies have investigated whether selective pressure is acting on a particular protein-coding gene in different genera/tribes of IR-loss clade. For instance, positive selection analyses suggested that *Lathyrus*, *Pisum* and *Vavilovia*, all belonging to tribe Fabeae, have undergone adaptive evolution in the *ycf*4 gene [15, 16]. Legume chloroplast genomes, and in particular IRLC, have regions with high mutation rates, including *rps*16-*acc*D-*psa*I-*ycf*4-*cem*A region. *rps*16 gene was lost from cpDNA in the common ancestor of the IR-loss clade [15]. *acc*D was completely absent in the *T.* subgenus *Trifolium* and has nuclear copies in *Medicago truncatula* and *Cicer arietinum *[22]. Three consecutive genes *psa*I-*ycf*4-*cem*A is situated in a local mutation hotspot and has been lost in some species of *Lathyrus *[15, 16].

### Phylogenetic relationships

With the use of the whole cp genome coding sequence from 28 representative species of the IR-loss clade, a highly consistent topology was recovered by ML and Bayesian analyses (Fig. 5). The monophyly of the IRLC was consistent with all previous studies [5, 14, 22, 42]. As shown in the previous studies, tribe Wisterieae together with tribe Glycyrrhizeae were the first diverging lineage as sister to the remaining taxa [19, 20, 42, 59, 60]. Tribes Caraganeae and Hedysareae were grouped together. Many previous studies showed that *Astragalus* was sister to the genus *Oxytropis* but recent study on the chloroplast phylogenomics of *Astragalus* reported that *Astragalus* is a monophyletic clade and *Oxytropis* is sister to the Coluteoid clade [42], which is in agreement with the present study. Cicereae + Trifolieae + Fabeae formed a well-supported clade. The results of the present study suggest that there is no conflict between the phylogeny made by whole cp genome and that inferred by individual gene datasets. Therefore, a phylogenetic reconstruction for IR-loss clade species studied here showed that plastid genome database will be a helpful resource for molecular phylogeny at the higher taxonomic level (generic to tribal rank).

## Conclusions

In this study, the complete plastome sequence of *O. gaubae* (122,688 bp) was determined. The gene contents and gene orientation of *O. gaubae* plastome are similar to those found in the plastid genome of other IRLC species. Comparison of plastomes across IRLC showed that the coding regions are more conserved than non-coding regions and IR is more conserved than LSC and SSC regions. The present study also analyzed genetic information in the IRLC plastomes including the distribution and location of repeat sequences and SSRs, codon usage, RNA editing prediction, hotspot regions and phylogenomic analysis. Moreover, we identified three hotspot genes (*ycf*1, *ycf*2, *clp*P) which provided sufficient genetic information for species identification and phylogenetic reconstruction of the IRLC species. Seven hypervariable regions including *ycf*1, *ycf*2, *clp*P, *acc*D and *ycf*4 (as the protein-coding regions) and *trn*L(UAA)-*trn*T(UGU) and *trn*T(GGU)-*trn*E(UUC) (as the intergenic regions) were also identified between *Onobrychis* species, which could be used to distinguish species. Finally, the data obtained from this study could provide a useful resource for further research on tribe Hedysareae and also IR-loss clade at the genomic scale.

## Methods

### Chloroplast DNA extraction and sequencing

The young leaves of *O. gaubae* were collected from the southern slopes of Alborz mountain range in Tehran, Iran. It was identified by Professor S. Kazempour-Osaloo. This species was preserved in the Tarbiat Modares University Herbarium (TMUH) (voucher code: 2016–1). Permission was not necessary for collecting the samples, which has not been included in the list of national key protected plants. The fresh leaves were immediately dried with silica gel for further DNA extraction. Our experimental research, including the collection of plant materials, are complies with institutional, national or international guidelines. Genomic DNA was extracted from dried leaves using a DNeasy Plant Kit (Qiagen) according to the manufacturer’s instructions. DNA quality and quantity were tested using 1% agarose gel electrophoresis and the resulting DNA was sequenced using the Illumina HiSeq-2000 platform at Iwate Biotechnology Research Center. The paired-end libraries were constructed according to the manufacturer’s protocol (Illumina Inc., San Diego, CA). In total, 43,189,861 paired-end reads each comprising 100-bp sequence were obtained.

### Genome assembly and annotation

Using the complete plastid genome of *Onobrychis viciifolia* (MW007721) as the reference, the paired-end reads of *O. gaubae* were filtered and assembled in to a complete plastome using Fast-Plast (https://github.com/mrmckain/Fast-Plast) [61]. Furthermore, we compared the chloroplast genome of *O. gaubae* with the complete chloroplast sequence of other Hedysareae species (*Hedysarum* and *Alhagi* species). Gaps in the cpDNA sequences were filled by PCR amplification and Sanger sequencing. The de novo assembled chloroplast genomes were annotated by GeSeq [62]. We used the online tRNAscan-SE service [63] to improve the identification of tRNA genes. To detect the number of matched reads and the depth of coverage, raw reads were remapped to the assembled plastomes with Bowtie2 [64] as implemented in Geneious v.9.0.2. The entire chloroplast genome sequences of *O. gaubae* was deposited in GenBank (Accession Number: LC647182).

To confirm the lack of IRa in the *O*. *gaubae*, it was surveyed by PCR and Sanger sequencing. A PCR strategy using primer pairs diagnostic for the presence or absence of the IRa region was conducted. The primer pairs were designed in either conserved *ndh*F and *psb*A, or *rps*19 and *rpl*2 protein coding sequences which are flanking the IR region boundaries, to allow the assessment of the presence or absence of the IRa region. The primer pairs used to detect the absence or presence of the IRa were: ndhF-F (5′-TATATGATTGGTCATATAATCG-3′) [65] and psbA-R (5′-GTTATGCATGAACGTAATGCTC-3′) [66]; rps19-F (5′-GTTCTGGACCAAGTTATT-3′) and rpl2-R (5′-ATTTGATTCTTCGTCGAC-3′) (designed in this study). The PCR amplification was carried out in the volume of 20 μl, containing 8 μl deionized water, 10 μl of the 2 × Taq DNA polymerase master mix Red (Amplicon), 0.5 μl of each primer (10 pmol/μl), and 1 μl of template DNA. PCR procedures for both regions were 2 min at 94 °C for predenaturation followed by 38 cycles of 1 min at 94 °C for denaturation, 1 min at 57 °C (when using ndhF-F and psbA-R primers) and 45 s at 56 °C (when using rps19-F and rpl2-R primers) for primer annealing and 50 s at 72 °C for primer extension, followed by a final primer extension of 5 min at 72 °C. PCR fragments were separated by electrophoresis in 1% agarose gels in 1 × TAE (pH = 8) buffer, stained with ethidium bromide and were photographed with a UV gel documentation system (UVItec, Cambridge, UK). PCR products along with the primers used for amplifcation were sent for Sanger sequencing at Macrogen (Seoul, South Korea).

### Codon usage

Codon usage was determined for all protein-coding genes. The codon usage analysis was performed in the web server Bioinformatics (https://www.bioinformatics.org/sms2/codon_usage.html). Furthermore, the relative synonymous codon usage (RSCU) values were determined with MEGA X [67], which was used to reveal the characteristics of the variation in synonymous codon usage.

### Characterization of repeat sequences

REPuter [68] was used to identify forward repeats, reverse sequences, complementary and palindromic sequences, with a minimal size of 30 bp, hamming distance of 3 and over 90% identity. Simple sequence repeats (SSRs) were detected using the microsatellite identification tool MISA (available online: http://pgrc.ipk-gatersleben.de/misa/misa.html). The minimum numbers of the SSR motifs were 10, 5, 4, 3, 3 and 3 for mono-, di-, tri-, tetra-, penta-, and hexanucleotide repeats, respectively.

### Divergent hotspots identification and synonymous (Ks) and non-synonymous (Ka) substitution rate analysis

To assess the nucleotide diversity (Pi) among the plastid genomes of the representative species of the IRLC, the whole chloroplast genome sequences were aligned using MAFFT [69] on XSEDE v.7.402 in CIPRES Science Gateway [70]. A sliding window analysis was conducted to determine the nucleotide diversity of the chloroplast genome using DnaSP v.6.12 software [37]. The window length was set to 800 bp and the step size was 200 bp. Furthermore, the protein-coding regions of the 28 chloroplast genomes were used to evaluate evolutionary rate variation within the IRLC. Thus, we aligned the 75 protein-coding genes separately using MAFFT and then estimated the synonymous (Ks) and non-synonymous (Ka) substitution rates, as well as their ratio (Ka/Ks) using DnaSP v.6.12 software.

### Genome comparison

To investigate divergence in chloroplast genomes, identity across the whole cp genomes was visualized using the mVISTA viewer in the Shuffle-LAGAN mode [71] among the 19 IRLC accessions using *Glycyrrhiza glabra* as the reference.

### Prediction of potential RNA editing sites

Thirty-five protein-coding genes of *O. gaubae* were used to predict potential RNA editing sites using the Predictive RNA Editor for Plants (PERP)-Cp web server (http://prep.unl.edu) [72] with a cutoff value of 0.8.

### Phylogenetic reconstruction

Seventy-five protein-coding genes were recorded from 28 species within IRLC, as well as from two outgroups [*Robinia pseudoacacia* L. and *Lotus japonicus* (Regel) K.Larsen]. The complete cp genome of *O*. *gaubae* obtained from this study and other 29 cp genomes downloaded from GenBank (Additional File 8: Table S12). The concatenated data were analyzed using maximum likelihood and Bayesian inference methodologies. Prior to maximum likelihood and Bayesian analyses, a general time reversible and gamma distribution (GTR + G) model was selected using the MrModeltest2.2 [73] under the Akaike Information Criteria (AIC )[74]. Maximum likelihood analyses were performed using the online phylogenetic software W-IQ-TREE [75] available at http://iqtree.cibiv.univie.ac.at. Nodes supports were calculated via rapid bootstrap analyses with 5000 replicates. Bayesian inference was performed using MrBayes v.3.2 in the CIPRES [70] with the following settings: Markov chain Monte Carlo simulations for 5,000,000 generations with four incrementally heated chains, starting from random trees and sampling one out of every 1,000 generations. The first 25% of the trees were regarded as burn-ins. The remaining trees were used to construct a 50% majority-rule consensus tree and to estimate posterior probabilities. Posterior probabilities (PP) > 0.95 were considered as significant support for a clade.

## Supplementary Information

**Additional file 1: Table S1.** Genes with intron in the O. gaubae chloroplast genome, including the exon and intron length. **Table S2.** Codon usage for *O*.* gaubae* chloroplast genome. **Table S3.** Codon usage for *O*.* viciifolia* chloroplast genome.

**Additional file 2: Table S4.** Putative preferred codons in the IRLC plastid genomes. RSCU = relative synonymous codon usage.

**Additional file 3: Table S5.** Forward, Reverse and Palindromic repeat sequences in the *O. gaubae* chloroplast genome. **Table S6.** Forward, Reverse and Palindromic repeat sequences in the *O. viciifolia* chloroplast genome. **Table S7.** Distribution of simple sequence repeat (SSR) in the *O. gaubae* chloroplast genome. **Table S8.** Distribution of simple sequence repeat (SSR) in the *O. viciifolia* chloroplast genome.

**Additional file 4: Figure S1.** Nucleotide variability (%) values between *O*. *gaubae* and *O*. *viciifolia* species.

**Additional file 5: Figure S2.** Sequence identity plot comparing the IRLC chloroplast genomes with *Glycyrrhiza* *glabra* as a reference.

**Additional file 6: Table S9.** The Ka, Ks and Ka/Ks ratio of IRLC chloroplast genome for individual genes and region.

**Additional file 7: Table S10.** Prediction of RNA editing sites in chloroplast genes of *O*. *gaubae*. **Table S11.** Prediction of RNA editing sites in chloroplast genes of *O*. *viciifolia*.

**Additional file 8: Table S12.** Accession number and sampled chloroplast genomes obtained from GenBank.

## Data Availability

Sequences used in this study are available from the National Center for Biotechnology Information (NCBI) (see Additional file 8: Table S12). Annotated sequence of plastome of *O*. *gaubae* were submitted to GenBank (http://getentry.ddbj.nig.ac.jp/) under LC647182 accession number. Sample of *O*. *gaubae* is saved at the Tarbiat Modares University Herbarium, Tehran, Iran.

## Abbreviations

SSR: Simple sequence repeat
cp: Chloroplast
IRs: Inverted repeats
LSC: Large single-copy
SSC: Small single-copy
IRLC: Inverted repeat lacking clade
ML: Maximum-likelihood
Ks: Synonymous substitution rates
Ka: Nonsynonymous substitution rates
RSCU: Relative synonymous codon usage
DnaSP: DNA sequence polymorphism
NCBI: National Center for Biotechnology
Pi: Nucleotide diversity/polymorphism
GTR: General time reversible
ITS: Internal transcribed spacer of ribosomal DNA
rRNA: Ribosomal RNA
tRNA: Transfer RNA
