# Supplementary material for: The complete chloroplast genome of Onobrychis gaubae (Fabaceae-Papilionoideae): comparative analysis with related IR-lacking clade species
Source: BMC Plant Biol. 2022 Feb 19;22:75. doi: 10.1186/s12870-022-03465-4 (PMC8858513; doi:10.1186/s12870-022-03465-4)
Supplement: Supplementary file 1 — Additional file 1: Table S1. Genes with intron in the O. gaubae chloroplast genome, including the exon and intron length. Table S2. Codon usage for O. gaubae chloroplast genome. Table S3. Codon usage for O. viciifolia chloroplast genome. [file 12870_2022_3465_MOESM1_ESM.docx]

**Table S1**. Genes with intron in the *O. gaubae* chloroplast genome, including the exon and intron length.

| **Gene name** | **Location** | **Exon I**  **(bp)** | **Intron I**  **(bp)** | **Exon II**  **(bp)** | **Intron II**  **(bp)** | **Exon III**  **(bp)** |
| --- | --- | --- | --- | --- | --- | --- |
| *ycf*3 | LSC | 114 | 706 | 227 | 736 | 154 |
| *atpF* | LSC | 144 | 703 | 409 | - | - |
| *rpoC1* | LSC | 431 | 712 | 1,622 | - | - |
| *petB* | LSC | 5 | 825 | 641 | - | - |
| *petD* | LSC | 7 | 713 | 474 | - | - |
| *rpl16* | LSC | 398 | 1,003 | 8 | - | - |
| *rpl2* | IR | 392 | 713 | 432 | - | - |
| *ndhB* | IR | 722 | 687 | 764 | - | - |
| *ndhA* | SSC | 550 | 1,236 | 540 | - | - |
| *clpP* | LSC | 367 | 600 | 228 | - | - |
| *rps*12 -5’ end | LSC | 117 | - | - | - | - |
| *rps*12 -3’ end | LSC | - | - | 258 | - | - |
| *trnK-UUU* | LSC | 35 | 2,495 | 37 | - | - |
| *trnC-ACA* | LSC | 36 | 611 | 36 | - | - |
| *trnL-UAA* | LSC | 37 | 542 | 50 | - | - |
| *trnA-UGC* | IR | 38 | 802 | 35 | - | - |
| *trnI-GAU* | IR | 35 | 788 | 37 | - | - |

**Table S2**. Codon usage for *O*. *gaubae* chloroplast genome.

| Amino Acid | Codon | Number | Fraction | Amino Acid | Codon | Number | Fraction |
| --- | --- | --- | --- | --- | --- | --- | --- |
| Ala | GCG  GCA  GCT  GCC | 159  360  387  213 | 0.14  0.32 0.35  0.19 | Asn | AAT  AAC | 1606  623 | 0.72  0.28 |
| Cys | TGT  TGC | 547  353 | 0.61  0.39 | Pro | CCG  CCA  CCT  CCC | 235  487  431  331 | 0.16  0.33  0.29  0.22 |
| Asp | GAT  GAC | 896  321 | 0.74  0.26 | Gln | CAG  CAA | 346  886 | 0.28  0.72 |
| Glu | GAG  GAA | 478  1194 | 0.29 0.71 | Arg | AGG  AGA  CGG  CGA  CGT  CGC | 455  847  258  387  273  163 | 0.19  0.36  0.11  0.16  0.11  0.07 |
| Phe | TTT  TTC | 2028  1052 | 0.66 0.34 | Ser | AGT  AGC  TCG  TCA  TCT  TCC | 579  360  433  715  865  590 | 0.16  0.10  0.12  0.20  0.24  0.17 |
| Gly | GGG  GGA  GGT  GGC | 428  674  466  246 | 0.24  0.37  0.26  0.14 | Thr | ACG  ACA  ACT  ACC | 284  601  581  406 | 0.15  0.32  0.31  0.22 |
| His | CAT  CAC | 645  274 | 0.70  0.30 | Val | GTG  GTA  GTT  GTC | 284  575  622  305 | 0.16  0.32  0.35  0.17 |
| Ile | ATA  ATT  ATC | 1406  1694  795 | 0.36  0.43  0.20 | Trp | TGG | 653 | 1.00 |
| Lys | AAG  AAA | 777  1952 | 0.28  0.72 | Tyr | TAT  TAC | 1431  557 | 0.72  0.28 |
| Leu | TTG  TTA  CTG  CTA  CTT  CTC | 808  1188  287  658  785  355 | 0.20  0.29  0.07  0.16  0.19  0.09 | End | TGA  TAG  TAA | 700  549  914 | 0.32  0.25  0.42 |
| Met | ATG | 703 | 1.00 |  |  |  |  |

**Table S3**. Codon usage for *O*. *viciifolia* chloroplast genome.

| Amino Acid | Codon | Number | Fraction | Amino Acid | Codon | Number | Fraction |
| --- | --- | --- | --- | --- | --- | --- | --- |
| Ala | GCG  GCA  GCT  GCC | 152  316  318  203 | 0.15  0.32 0.32  0.21 | Asn | AAT  AAC | 1595  594 | 0.73  0.27 |
| Cys | TGT  TGC | 506  302 | 0.63  0.37 | Pro | CCG  CCA  CCT  CCC | 239  478  396  332 | 0.17  0.33  0.27  0.23 |
| Asp | GAT  GAC | 871  322 | 0.73  0.27 | Gln | CAG  CAA | 364  872 | 0.29  0.71 |
| Glu | GAG  GAA | 503  1166 | 0.30 0.70 | Arg | AGG  AGA  CGG  CGA  CGT  CGC | 434  780  274  429  271  163 | 0.18  0.33  0.12  0.18  0.12  0.07 |
| Phe | TTT  TTC | 1920  1032 | 0.65 0.35 | Ser | AGT  AGC  TCG  TCA  TCT  TCC | 539  319  385  676  838  554 | 0.16  0.10  0.12  0.20  0.25  0.17 |
| Gly | GGG  GGA  GGT  GGC | 398  631  415  207 | 0.24  0.38  0.25  0.13 | Thr | ACG  ACA  ACT  ACC | 272  498  463  388 | 0.17  0.31  0.29  0.24 |
| His | CAT  CAC | 696  286 | 0.71  0.29 | Val | GTG  GTA  GTT  GTC | 325  608  646  325 | 0.17  0.32  0.34  0.17 |
| Ile | ATA  ATT  ATC | 1353  1537  748 | 0.37  0.42  0.21 | Trp | TGG | 614 | 1.00 |
| Lys | AAG  AAA | 773  1899 | 0.29  0.71 | Tyr | TAT  TAC | 1477  578 | 0.72  0.28 |
| Leu | TTG  TTA  CTG  CTA  CTT  CTC | 909  1089  366  651  838  436 | 0.21  0.25  0.09  0.15  0.20  0.10 | End | TGA  TAG  TAA | 673  711  967 | 0.29  0.30  0.41 |
| Met | ATG | 724 | 1.00 |  |  |  |  |
