# Supplementary material for: The complete chloroplast genome of Onobrychis gaubae (Fabaceae-Papilionoideae): comparative analysis with related IR-lacking clade species
Source: BMC Plant Biol. 2022 Feb 19;22:75. doi: 10.1186/s12870-022-03465-4 (PMC8858513; doi:10.1186/s12870-022-03465-4)
Supplement: Supplementary file 3 — Additional file 3: Table S5. Forward, Reverse and Palindromic repeat sequences in the O. gaubae chloroplast genome. Table S6. Forward, Reverse and Palindromic repeat sequences in the O. viciifolia chloroplast genome. Table S7. Distribution of simple sequence repeat (SSR) in the O. gaubae chloroplast genome. Table S8. Distribution of simple sequence repeat (SSR) in the O. viciifolia chloroplast genome. [file 12870_2022_3465_MOESM3_ESM.docx]

**Table S5**. Forward, Reverse and Palindromic repeat sequences in the *O. gaubae* chloroplast genome.

| No. | Type | Location | Size  (in bp) | Location | Region |
| --- | --- | --- | --- | --- | --- |
| 1 | Forward | 1820 | 91 | *trn*K*-*UUU | LSC |
| 2 | Forward | 67360 | 179 | *rps*12 | LSC |
| 3 | Forward | 94201 | 160 | *rps*12 | IRB |
| 4 | Forward | 67402 | 137 | *rps*12 | LSC |
| 5 | Forward | 94098 | 120 | IGS (*rps*7 – *rps*12) | IRB |
| 6 | Forward | 21109 | 81 | *psa*A | LSC |
| 7 | Forward | 23344 | 80 | *psa*B | LSC |
| 8 | Forward | 28487 | 53 | *psb*C | LSC |
| 9 | Forward | 56208 | 61 | *acc*D | SSC |
| 10 | Palindrome | 79135 | 50 | *rpl*16 (intron) | LSC |
| 11 | Palindrome | 117597 | 51 | IGS (*psa*C-*ndh*D) | SSC |
| 12 | Forward | 57511 | 50 | IGS (*acc*D - *psa*I) | LSC |
| 13 | Forward | 57837 | 52 | *ycf*4 | LSC |
| 14 | Palindrome | 19410 | 42 | IGS (*ycf*3 - *psa*A) | LSC |
| 15 | Palindrome | 51984 | 45 | *trn*R-UCU | LSC |
| 16 | Forward | 57509 | 44 | IGS (*acc*D - *psa*I) | LSC |
| 17 | Palindrome | 121048 | 40 | IGS (*trn*L-UAG – *rpl*32) | SSC |
| 18 | Forward | 94405 | 42 | *rps*12 (intron) | IRB |
| 19 | Forward | 57842 | 47 | IGS (*psa*I - *ycf*4) | LSC |
| 20 | Forward | 56215 | 40 | IGS (*trn*Q-UUG - *acc*D) | LSC |
| 21 | Palindrome | 114295 | 40 | *ndh*A (intron) | SSC |
| 22 | Forward | 11303 | 35 | IGS (*trn*V-UAC - *ndh*C) | LSC |
| 23 | Palindrome | 48703 | 40 | IGS (*atp*H - *atp*F) | LSC |
| 24 | Forward | 56272 | 40 | *acc*D | LSC |
| 25 | Palindrome | 119359 | 40 | IGS (*ndh*D - *ccs*A) | SSC |
| 26 | Palindrome | 18170 | 39 | *ycf*3 (intron) | LSC |
| 27 | Forward | 109415 | 32 | *ycf*1 | IRB |
| 28 | Palindrome | 31524 | 38 | IGS (*trn*Y-GUA – *trn*D-GUC) | LSC |
| 29 | Reverse | 115558 | 37 | IGS (*ndh*I - *ndh*G) | SSC |
| 30 | Palindrome | 10330 | 30 | IGS (*atp*E – *trn*M-CAU) | LSC |
| 31 | Forward | 78102 | 30 | IGS (*rps*8 – *rpl*14) | LSC |
| 32 | Forward | 96349 | 30 | IGS (*trn*V-GAC – *rrn*16) | IRB |
| 33 | Palindrome | 108228 | 30 | *ycf*1 | IRB |
| 34 | Palindrome | 79150 | 36 | *rpl*16 (intron) | LSC |
| 35 | Forward | 56222 | 33 | *acc*D | LSC |
| 36 | Forward | 56279 | 33 | *acc*D | LSC |
| 37 | Palindrome | 17058 | 35 | IGS (*rps*4 – *trn*S-GGA) | LSC |
| 38 | Forward | 18171 | 35 | *ycf*3 (intron) | LSC |
| 39 | Palindrome | 84540 | 35 | *ycf*2 | IRB |
| 40 | Palindrome | 110607 | 37 | IGS (*ycf*1 – *rps*15) | SSC |
| 41 | Forward | 56336 | 33 | *acc*D | LSC |
| 42 | Forward | 72528 | 35 | *pet*B (intron) | LSC |
| 43 | Palindrome | 94408 | 35 | *rps*12 (intron) | IRB |
| 44 | Forward | 21168 | 32 | *psa*A | LSC |
| 45 | Forward | 95113 | 32 | IGS (*rps*12 – *trn*V-GAC) | IRB |
| 46 | Palindrome | 124214 | 34 | IGS (*ndh*F – *trn*H-GUG) | SSC |
| 47 | Reverse | 61396 | 31 | *psb*J | LSC |
| 48 | Forward | 56312 | 33 | *acc*D | LSC |
| 49 | Forward | 56279 | 33 | *acc*D | LSC |
| 50 | Palindrome | 79150 | 33 | *rpl*16 (intron) | LSC |

P: means palindromic, F: means forward, R: means reverse and IGS: means intergenic spacer.

**Table S6**. Forward, Reverse and Palindromic repeat sequences in the *O. viciifolia* chloroplast genome.

| No. | Type | Location | Size  (in bp) | Location | Region |
| --- | --- | --- | --- | --- | --- |
| 1 | Forward | 87140 | 91 | *ycf*2 | IRB |
| 2 | Forward | 19780 | 91 | *psa*A | LSC |
| 3 | Forward | 88065 | 79 | IGS (*trn*I-CAU – *ndh*B) | IRB |
| 4 | Palindromic | 81836 | 56 | IGS (*trn*L-CAA – *ycf*2) | IRB |
| 5 | Forward | 54181 | 61 | *acc*D | LSC |
| 6 | Palindrome | 12446 | 56 | IGS (*ndh*J – *trn*F-GAA) | LSC |
| 7 | Forward | 55509 | 54 | IGS (*acc*D – *psa*I) | LSC |
| 8 | Palindrome | 76615 | 50 | *rpl*16 | LSC |
| 9 | Palindrome | 81125 | 46 | IGS (*rpl*23 – *trn*L-CAA) | IRB |
| 10 | Palindrome | 88483 | 49 | IGS (*trn*I-CAU – *ndh*B) | IRB |
| 11 | Palindrome | 18060 | 42 | IGS (*ycf*3 - *psa*A) | LSC |
| 12 | Palindrome | 118571 | 42 | IGS (*trn*L-UAG – *rpl*32) | SSC |
| 13 | Forward | 55519 | 44 | IGS (*acc*D – *psa*I) | LSC |
| 14 | Palindrome | 116875 | 38 | IGS (*ndh*D - *ccs*A) | SSC |
| 15 | Forward | 54188 | 40 | *acc*D | LSC |
| 16 | Palindrome | 91862 | 40 | IGS (*rps*12 – *trn*V-GAC) | IRB |
| 17 | Palindrome | 49930 | 45 | *trn*R-UCU | LSC |
| 18 | Forward | 65558 | 38 | IGS (*rps*12 – *clp*P) | LSC |
| 19 | Forward | 76625 | 41 | *rpl*16 | LSC |
| 20 | Palindrome | 47354 | 40 | IGS (*atp*H - *atp*F) | LSC |
| 21 | Forward | 54245 | 40 | *acc*D | LSC |
| 22 | Palindrome | 16776 | 39 | *ycf*3 | LSC |
| 23 | Forward | 106871 | 32 | *ycf*1 | IRB |
| 24 | Palindrome | 30145 | 38 | IGS (*trn*Y-GUA – *trn*D-GUC) | LSC |
| 25 | Forward | 65729 | 31 | *clp*P | LSC |
| 26 | Forward | 802 | 39 | *psb*A | LSC |
| 27 | Palindrome | 31272 | 39 | IGS (*pet*N – *trn*C-GCA) | LSC |
| 28 | Forward | 75582 | 30 | IGS (*rps*8 – *rpl*14) | LSC |
| 29 | Forward | 87201 | 30 | *ycf*2 | IRB |
| 30 | Forward | 93798 | 30 | IGS (*trn*V-GAC – *rrn*16) | IRB |
| 31 | Palindrome | 105687 | 30 | *ycf*1 | IRB |
| 32 | Forward | 54195 | 33 | *acc*D | LSC |
| 33 | Forward | 54228 | 33 | *acc*D | LSC |
| 34 | Forward | 16777 | 38 | *ycf*3 (intron) | LSC |
| 35 | Palindrome | 15668 | 35 | IGS (*rps*4 – *trn*S-GGA) | LSC |
| 36 | Palindrome | 70012 | 35 | *pet*B (intron) | LSC |
| 37 | Palindrome | 153 | 31 | IGS (*trn*H-GUG – *psb*A) | LSC |
| 38 | Forward | 54309 | 33 | *acc*D | LSC |
| 39 | Forward | 111785 | 35 | *ndh*A (intron) | SSC |
| 40 | Forward | 19839 | 32 | *psa*A | LSC |
| 41 | Forward | 92570 | 32 | IGS (*rps*12 – *trn*V-GAC) | IRB |
| 42 | Reverse | 59520 | 31 | *psb*J | LSC |
| 43 | Forward | 93775 | 31 | IGS (*trn*V-GAC – *rrn*16) | IRB |
| 44 | Forward | 54285 | 33 | *acc*D | LSC |
| 45 | Forward | 54252 | 33 | *acc*D | LSC |
| 46 | Reverse | 81210 | 30 | IGS (*trn*L-CAA – *ycf*2) | IRB |
| 47 | Forward | 92622 | 30 | IGS (*rps*12 – *trn*V-GAC) | IRB |
| 48 | Forward | 25572 | 32 | *trn*S-UGA | LSC |
| 49 | Palindrome | 87224 | 32 | *ycf2* | IRB |
| 50 | Forward | 106953 | 31 | *ycf1* | IRB |

P: means palindromic, F: means forward, R: means reverse and IGS: means intergenic spacer.

**Table S7**. Distribution of simple sequence repeat (SSR) in the O. gaubae chloroplast genome.

| No. SSR | SSR type | SSR | Size | Start | End | Location |
| --- | --- | --- | --- | --- | --- | --- |
| 1 | p1 | (T)11 | 11 | 2072 | 2082 | LSC |
| 2 | p1 | (T)11 | 11 | 4351 | 4361 | LSC |
| 3 | p1 | (A)10 | 10 | 5656 | 5665 | LSC |
| 4 | p2 | (TA)5 | 10 | 6070 | 6079 | LSC |
| 5 | p1 | (T)10 | 10 | 7872 | 7881 | LSC |
| 6 | c | (A)11cggatatattttaatatgaaaataactcctacaacttctgatactgaggtttctgcacttg(A)10 | 82 | 8359 | 8440 | LSC |
| 7 | p2 | (TA)5 | 10 | 12950 | 12959 | LSC |
| 8 | p2 | (AT)5 | 10 | 13941 | 13950 | LSC |
| 9 | p1 | (A)10 | 10 | 14142 | 14151 | LSC |
| 10 | p1 | (T)10 | 10 | 15984 | 15993 | LSC |
| 11 | p2 | (AT)7 | 14 | 25487 | 25500 | LSC |
| 12 | c | (AT)7taacatagataattggaatattctattccaatttttctatttttttctta(AT)5 | 74 | 25990 | 26063 | LSC |
| 13 | p2 | (AT)7 | 14 | 30477 | 30490 | LSC |
| 14 | p1 | (A)10 | 10 | 30703 | 30712 | LSC |
| 15 | p1 | (T)10 | 10 | 31068 | 31077 | LSC |
| 16 | p1 | (A)11 | 11 | 32054 | 32064 | LSC |
| 17 | p3 | (ATA)4 | 12 | 33557 | 33568 | LSC |
| 18 | p2 | (AT)5 | 10 | 37629 | 37638 | LSC |
| 19 | p4 | (AATA)3 | 12 | 39146 | 39157 | LSC |
| 20 | p2 | (AT)5 | 10 | 42137 | 42146 | LSC |
| 21 | p1 | (A)11 | 11 | 43623 | 43633 | LSC |
| 22 | p1 | (A)10 | 10 | 45442 | 45451 | LSC |
| 23 | c | (T)10caa(T)11gt(A)10 | 36 | 45777 | 45812 | LSC |
| 24 | p1 | (A)10 | 10 | 46051 | 46060 | LSC |
| 25 | p1 | (A)13 | 13 | 47712 | 47724 | LSC |
| 26 | p1 | (T)10 | 10 | 48560 | 48569 | LSC |
| 27 | p1 | (A)10 | 10 | 50159 | 50168 | LSC |
| 28 | p2 | (AT)5 | 10 | 51902 | 51911 | LSC |
| 29 | p5 | (AATCT)3 | 15 | 52685 | 52699 | LSC |
| 30 | p1 | (T)11 | 11 | 53281 | 53291 | LSC |
| 31 | c | (A)11cgacagagaaagagaatagattctcttctatttcacattatgttttctttgatactaagtttctaagaaataaagtgatttctaggaaagc(A)10 | 112 | 53922 | 54033 | LSC |
| 32 | p1 | (T)10 | 10 | 54167 | 54176 | LSC |
| 33 | p3 | (TTA)4 | 12 | 54695 | 54706 | LSC |
| 34 | p1 | (T)10 | 10 | 57117 | 57126 | LSC |
| 35 | p2 | (AT)5 | 10 | 57379 | 57388 | LSC |
| 36 | c | (T)12caaaattaaaaaaggaaagaactaacttccgaattacaaataatt(A)11 | 68 | 58606 | 58673 | LSC |
| 37 | p1 | (T)11 | 11 | 60875 | 60885 | LSC |
| 38 | p2 | (AT)5 | 10 | 61607 | 61616 | LSC |
| 39 | p3 | (TTC)4 | 12 | 62512 | 62523 | LSC |
| 40 | p1 | (A)11 | 11 | 63019 | 63029 | LSC |
| 41 | p1 | (T)10 | 10 | 64013 | 64022 | LSC |
| 42 | p2 | (AT)5 | 10 | 64163 | 64172 | LSC |
| 43 | p1 | (A)10 | 10 | 64609 | 64618 | LSC |
| 44 | p1 | (A)15 | 15 | 65807 | 65821 | LSC |
| 45 | p1 | (A)10 | 10 | 68328 | 68337 | LSC |
| 46 | p1 | (T)10 | 10 | 77958 | 77967 | LSC |
| 47 | c | (A)11tatt(TA)6 | 27 | 79203 | 79229 | LSC |
| 48 | p1 | (A)11 | 11 | 81195 | 81205 | LSC |
| 49 | p1 | (T)10 | 10 | 81464 | 81473 | LSC |
| 50 | p2 | (TA)5 | 10 | 82238 | 82247 | IRB |
| 51 | p3 | (AAT)4 | 12 | 83763 | 83774 | IRB |
| 52 | c | (ATTA)3aataaatactttctttta(TAT)4 | 42 | 90712 | 90753 | IRB |
| 53 | p1 | (T)10 | 10 | 94068 | 94077 | IRB |
| 54 | p2 | (AT)7 | 14 | 95536 | 95549 | IRB |
| 55 | c | (AT)7taaaggaactgtaaattctattaaaatatatatgtatatattcaa(AT)5 | 69 | 96346 | 96414 | IRB |
| 56 | p1 | (T)10 | 10 | 98687 | 98696 | IRB |
| 57 | p4 | (AGGT)3 | 12 | 102015 | 102026 | IRB |
| 58 | p2 | (AG)5 | 10 | 103810 | 103819 | IRB |
| 59 | c | (TA)8gatagaac(TA)5 | 34 | 104796 | 104829 | IRB |
| 60 | p1 | (T)10 | 10 | 106548 | 106557 | IRB (*ycf*1) |
| 61 | p1 | (A)10 | 10 | 106668 | 106677 | IRB (*ycf*1) |
| 62 | p1 | (A)10 | 10 | 107264 | 107273 | IRB (*ycf*1) |
| 63 | c | (A)12gatccactg(A)10tgtg(A)11 | 46 | 107624 | 107669 | IRB  (*ycf*1) |
| 64 | p2 | (AT)5 | 10 | 110649 | 110658 | SSC |
| 65 | p3 | (TAA)4 | 12 | 110865 | 110876 | SSC |
| 66 | c | (AAAT)3aaaattatttataattaaatagaattcttaataaaaaaatcatataaatcaaatcatcttctatattatttgattc(TA)5 | 98 | 115472 | 115569 | SSC |
| 67 | p1 | (T)11 | 11 | 116603 | 116613 | SSC |
| 68 | p4 | (TATT)3 | 12 | 118965 | 118976 | SSC |
| 69 | p2 | (AT)6 | 12 | 120397 | 120408 | SSC |
| 70 | p1 | (T)11 | 11 | 121311 | 121321 | SSC |

**Table S8**. Distribution of simple sequence repeat (SSR) in the O. viciifolia chloroplast genome.

| No. SSR | SSR type | SSR | Size | Start | End | Location |
| --- | --- | --- | --- | --- | --- | --- |
| 1 | p1 | (T)11 | 11 | 2074 | 2084 | LSC |
| 2 | p4 | (AAAT)3 | 12 | 3446 | 3457 | LSC |
| 3 | p2 | (TA)5 | 10 | 4535 | 4544 | LSC |
| 4 | p1 | (T)13 | 13 | 6304 | 6316 | LSC |
| 5 | p1 | (A)10 | 10 | 6419 | 6428 | LSC |
| 6 | c | (A)12cggatatattttaatatgagaataactcctacaacttctgatactgaggtttctgcacttg(A)10 | 83 | 6789 | 6871 | LSC |
| 7 | p1 | (T)10 | 10 | 9119 | 9128 | LSC |
| 8 | c | (TA)6aatatataaagacttttagtagtagaaagaataaataaatttctattcgaatattattctatat(TA)7 | 90 | 9856 | 9945 | LSC |
| 9 | p2 | (TA)5 | 10 | 11474 | 11483 | LSC |
| 10 | c | (TATC)3ccccaattcattaaaatgaattgggg(GATA)3 | 50 | 12450 | 12499 | LSC |
| 11 | p1 | (A)11 | 11 | 12651 | 12661 | LSC |
| 12 | c | (TA)6ttatctatatctatatat(TA)5 | 40 | 13707 | 13746 | LSC |
| 13 | c | (T)10acggatctcagtcttaggagaaagatacattcttcggagagaaaggtaaaatttttg(A)10 | 77 | 16154 | 16230 | LSC |
| 14 | p1 | (A)10 | 10 | 17178 | 17187 | LSC |
| 15 | p1 | (T)11 | 11 | 18186 | 18196 | LSC |
| 16 | c | (AAG)4agatactactatagtatcacagtcaactctaaaaaatcttttttaaaagagaaatagaatatatattcta(AT)6 | 94 | 24094 | 24187 | LSC |
| 17 | c | (AT)7taacatagataattggaatattctattccaatttttctatttttttctta(AT)5aactatatatttctcttttttttagttttactatatatcatattctatttagtataaaagataagatttttc(A)11 | 157 | 24665 | 24821 | LSC |
| 18 | p1 | (A)11 | 11 | 28905 | 28915 | LSC |
| 19 | p2 | (AT)7 | 14 | 29092 | 29105 | LSC |
| 20 | p1 | (T)10 | 10 | 29687 | 29696 | LSC |
| 21 | p4 | (TATG)3 | 12 | 30513 | 30524 | LSC |
| 22 | p1 | (A)11 | 11 | 30680 | 30690 | LSC |
| 23 | p1 | (A)11 | 11 | 33278 | 33288 | LSC |
| 24 | p2 | (AT)5 | 10 | 36229 | 36238 | LSC |
| 25 | p4 | (AATA)3 | 12 | 37751 | 37762 | LSC |
| 26 | p2 | (AT)10 | 10 | 40811 | 40820 | LSC |
| 27 | p1 | (A)13 | 13 | 42291 | 42303 | LSC |
| 28 | p1 | (A)10 | 10 | 44113 | 44122 | LSC |
| 29 | c | (T)10caa(T)11 | 24 | 44437 | 44460 | LSC |
| 30 | p1 | (A)10 | 10 | 44711 | 44720 | LSC |
| 31 | p1 | (A)13 | 13 | 46364 | 46376 | LSC |
| 32 | p1 | (T)10 | 10 | 47211 | 47220 | LSC |
| 33 | p2 | (AT)5 | 10 | 49848 | 49857 | LSC |
| 34 | p5 | (AATCT)3 | 15 | 50620 | 50634 | LSC |
| 35 | c | (T)10caaatcaaatacattcaattcaaataaatcattgttatctaggattcatggg(A)11 | 73 | 51233 | 51305 | LSC |
| 36 | p1 | (T)10 | 10 | 51669 | 51678 | LSC |
| 37 | p1 | (A)10 | 10 | 51994 | 52003 | LSC |
| 38 | p1 | (T)10 | 10 | 52137 | 52146 | LSC |
| 39 | p3 | (TTA)4 | 12 | 52665 | 52676 | LSC |
| 40 | p1 | (T)10 | 10 | 55090 | 55099 | LSC |
| 41 | p2 | (AT)5 | 10 | 55352 | 55361 | LSC |
| 42 | p1 | (T)12 | 12 | 56652 | 56663 | LSC |
| 43 | c | (TA)5ttgtgtgtgaagaagactgttttactttgtg(T)10 | 51 | 58876 | 58926 | LSC |
| 44 | p2 | (AT)5 | 10 | 59730 | 59739 | LSC |
| 45 | c | (CATT)3attatcttcggactacaaactgagaatcaattaaaatgcgaatcgaatgaattggag(TTC)4 | 81 | 60570 | 60650 | LSC |
| 46 | p1 | (T)10 | 10 | 62138 | 62147 | LSC |
| 47 | p2 | (AT)5 | 10 | 62288 | 62297 | LSC |
| 48 | p1 | (A)15 | 15 | 63918 | 63932 | LSC |
| 49 | p1 | (T)10 | 10 | 65059 | 65068 | LSC |
| 50 | c | (AAT)4actacagtttgtgatcatcagttttggtttcttctttttacatagaaatataccttttgtaaacatcgacttatc(CTT)4 | 99 | 65640 | 65738 | LSC |
| 51 | p3 | (CTC)4 | 12 | 66328 | 66339 | LSC |
| 52 | p1 | (T)11 | 11 | 75200 | 75210 | LSC |
| 53 | p1 | (T)10 | 10 | 75438 | 75447 | LSC |
| 54 | c | (A)11tatt(TA)6 | 27 | 76683 | 76709 | LSC |
| 55 | p4 | (TTTC)4 | 16 | 77422 | 77437 | LSC |
| 56 | p1 | (A)11 | 11 | 78695 | 78705 | LSC |
| 57 | p1 | (T)10 | 10 | 78964 | 78973 | LSC |
| 58 | p1 | (T)11 | 11 | 79109 | 79119 | IRB |
| 59 | p1 | (T)10 | 10 | 81042 | 81051 | IRB |
| 60 | p3 | (AAT)4 | 12 | 81252 | 81263 | IRB |
| 61 | p3 | (TCA)5 | 15 | 83808 | 83822 | IRB |
| 62 | c | (ATTA)3aataaatactttctttta(TAT)4 | 42 | 88199 | 88240 | IRB |
| 63 | p1 | (T)10 | 10 | 91534 | 91543 | IRB |
| 64 | p2 | (AT)7 | 14 | 92993 | 93006 | IRB |
| 65 | c | (AT)7taaaggaactgtaaattctattaaaatatatatgtatatattcaa(AT)5 | 69 | 93795 | 93863 | IRB |
| 66 | p1 | (T)10 | 10 | 96137 | 96146 | IRB |
| 67 | p4 | (AGGT)3 | 12 | 99475 | 99486 | IRB |
| 68 | p2 | (AG)5 | 10 | 101275 | 101284 | IRB |
| 69 | c | (TA)8gatagaac(TA)5 | 34 | 102261 | 102294 | IRB |
| 70 | p1 | (T)10 | 10 | 104010 | 104019 | IRB (*ycf*1) |
| 71 | p1 | (A)12 | 12 | 105092 | 105103 | IRB (*ycf*1) |
| 72 | p1 | (A)11 | 11 | 107960 | 107970 | IRB (*ycf*1) |
| 73 | p2 | (TA)7 | 14 | 108169 | 108182 | SSC |
| 74 | p1 | (A)14 | 14 | 108397 | 108410 | SSC |
| 75 | p4 | (AAAT)3 | 12 | 112960 | 112971 | SSC |
| 76 | p1 | (T)15 | 15 | 113354 | 113368 | SSC |
| 77 | p4 | (TATT)3 | 12 | 116474 | 116485 | SSC |
| 78 | p2 | (AT)5 | 10 | 116890 | 116899 | SSC |
| 79 | p2 | (AT)6 | 12 | 117919 | 117930 | SSC |
| 80 | c | (AT)5agaaaaaatgaatagtaacgataagacgataagttactattaataataagtatagtatagtaatttattataattaaaa(AT)6 | 101 | 118351 | 118451 | SSC |
| 81 | p2 | (TA)5 | 10 | 118588 | 118597 | SSC |
| 82 | p1 | (T)11 | 11 | 118829 | 118839 | SSC |
| 83 | p1 | (A)10 | 10 | 120938 | 120947 | SSC |
