# Supplementary material for: The complete chloroplast genome of Onobrychis gaubae (Fabaceae-Papilionoideae): comparative analysis with related IR-lacking clade species
Source: BMC Plant Biol. 2022 Feb 19;22:75. doi: 10.1186/s12870-022-03465-4 (PMC8858513; doi:10.1186/s12870-022-03465-4)
Supplement: Supplementary file 5 — Additional file 5: Figure S2. Sequence identity plot comparing the IRLC chloroplast genomes with Glycyrrhiza glabra as a reference. [file 12870_2022_3465_MOESM5_ESM.pdf]

Figure S2.

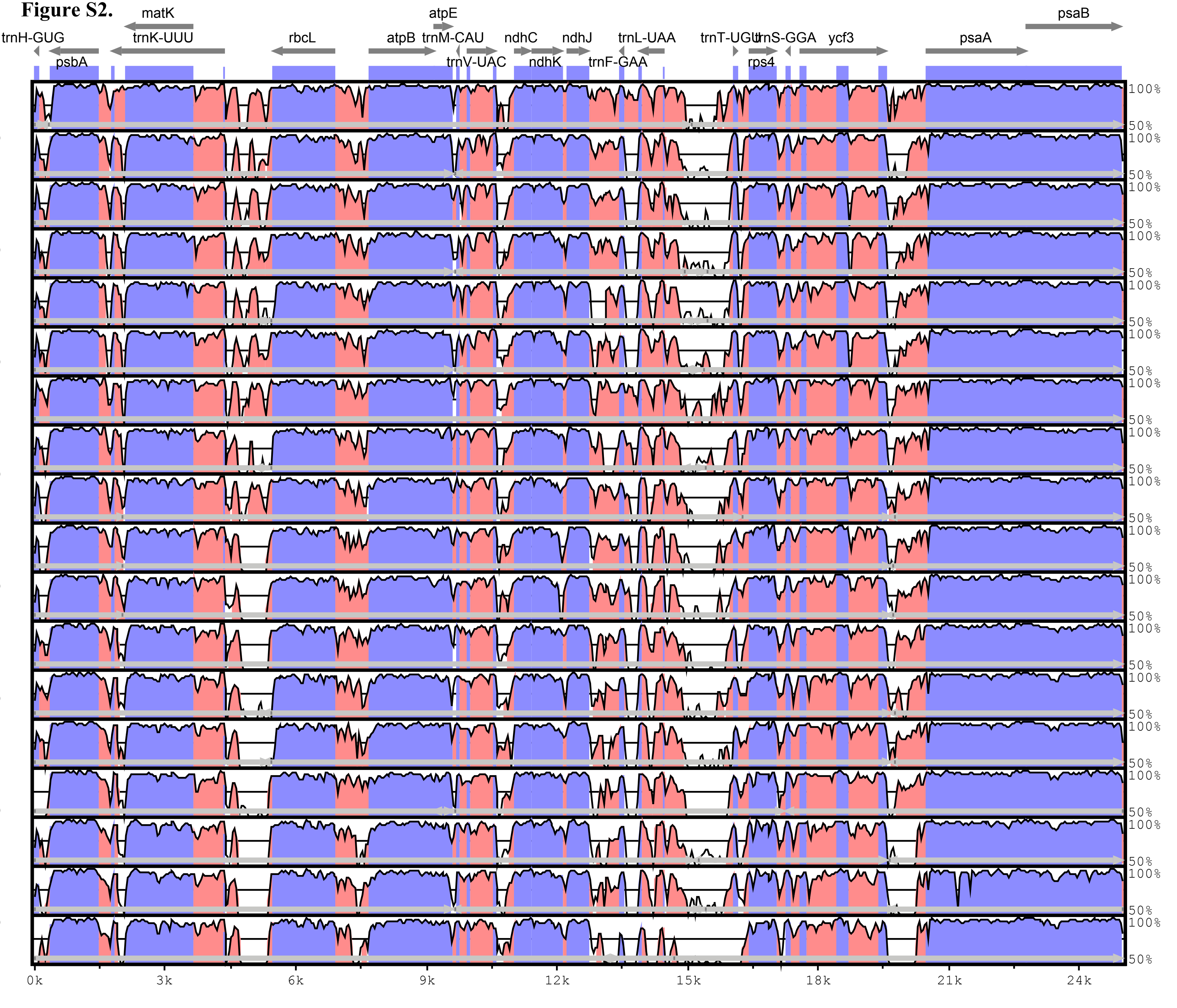

matK
atpE
psaB
trnH-GUG
trnK-UUU
rbcL
atpB
trnM-CAU
ndhC
ndhJ
trnL-UAA
trnT-UGU
trnS-GGA
ycf3
psaA
psbA
trnV-UAC
ndhK
trnF-GAA
rps4
100%
50%
0k
3k
6k
9k
12k
15k
18k
21k
24k

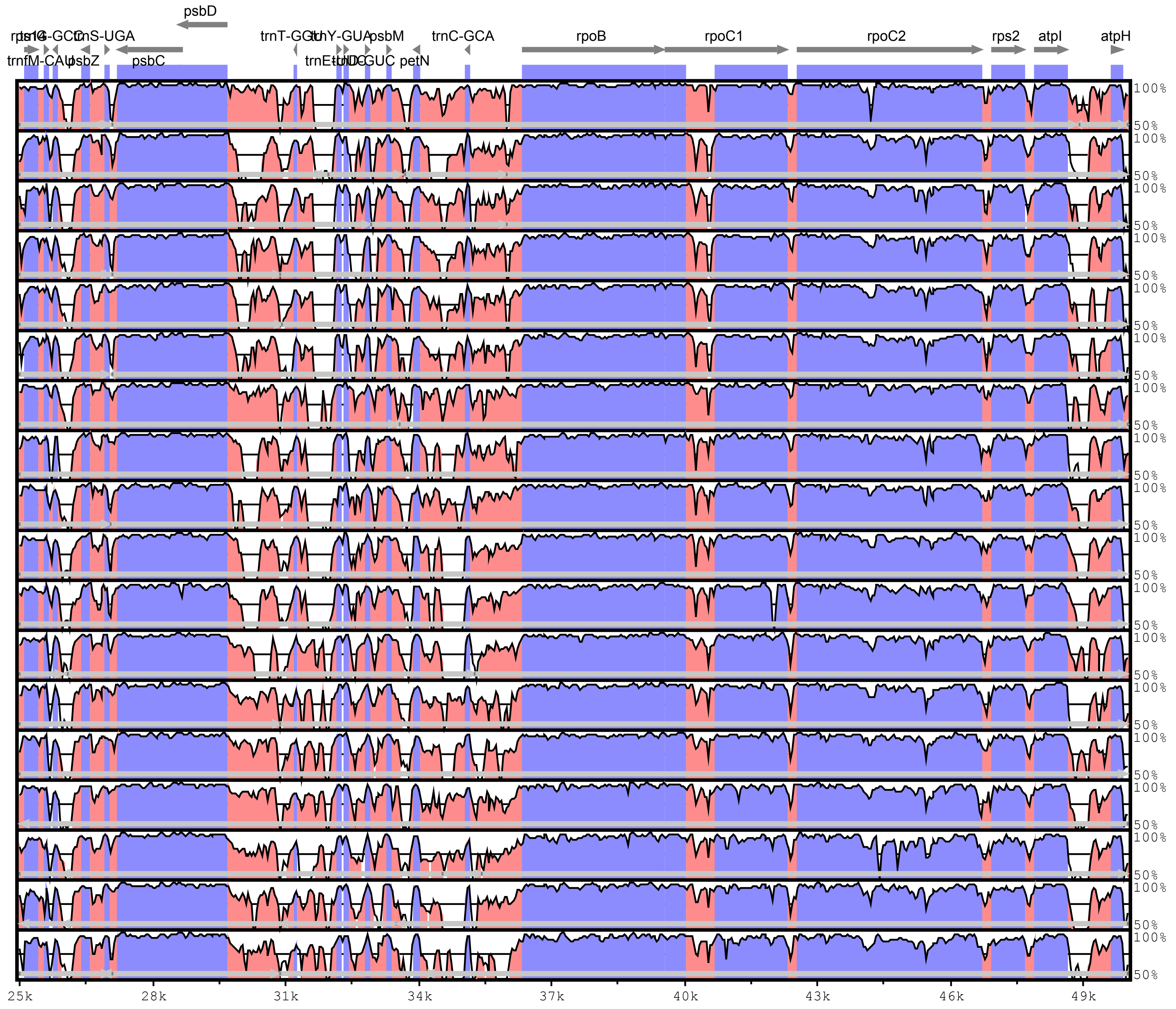

psbD
trnS-UGA
trnY-GUA
psbM
trnC-GCA
rpoB
rpoC1
rpoC2
rps2
atpI
atpH
psbZ
psbC
petN
100%
50%
25k
28k
31k
34k
37k
40k
43k
46k
49k

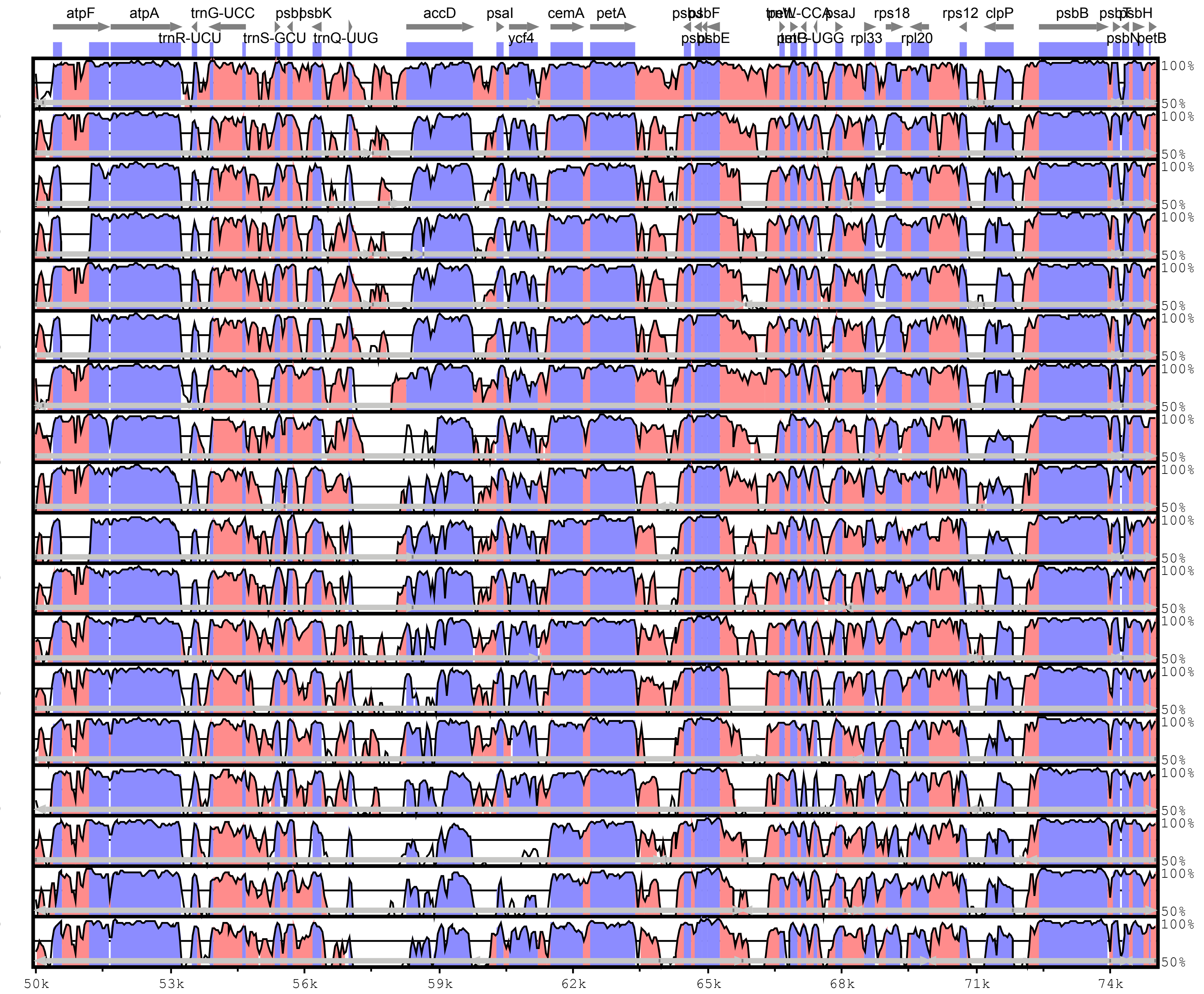

atpF
atpA
trnG-UCC
trnR-UCU
trnS-GCU
trnQ-UUG
psbK
accD
psaI
ycf4
cemA
petA
psbF
psbE
psaJ
rpl33
rps18
rpl20
rps12
clpP
psbB
psbH
petB
100%
50%
50k
53k
56k
59k
62k
65k
68k
71k
74k

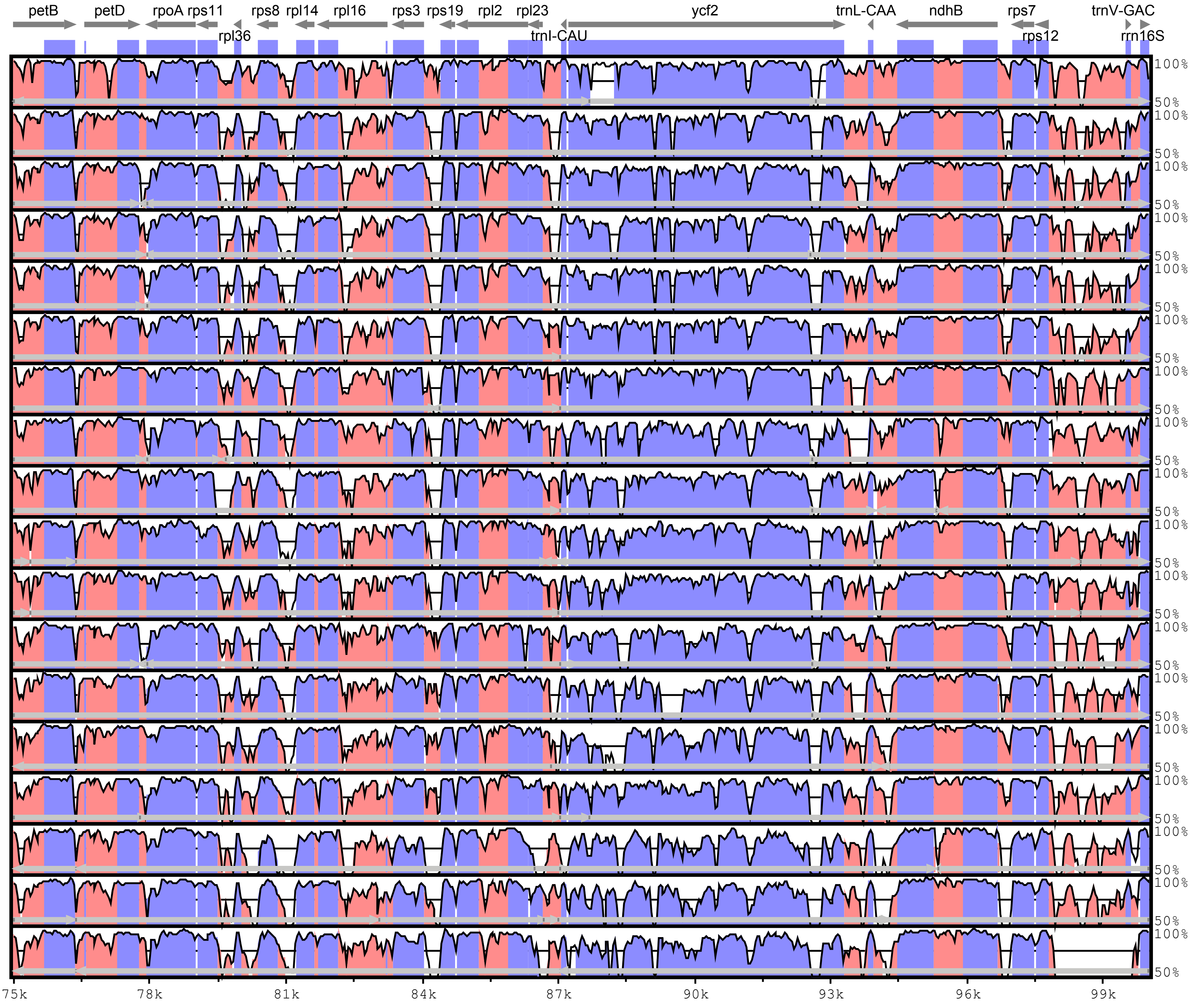
petB
petD
rpoA
rps11
rpl36
rps8
rpl14
rpl16
rps3
rps19
rpl2
rpl23
trnI-CAU
ycf2
trnL-CAA
ndhB
rps7
rps12
trnV-GAC
rrn16S
100%
50%
75k
78k
81k
84k
87k
90k
93k
96k
99k

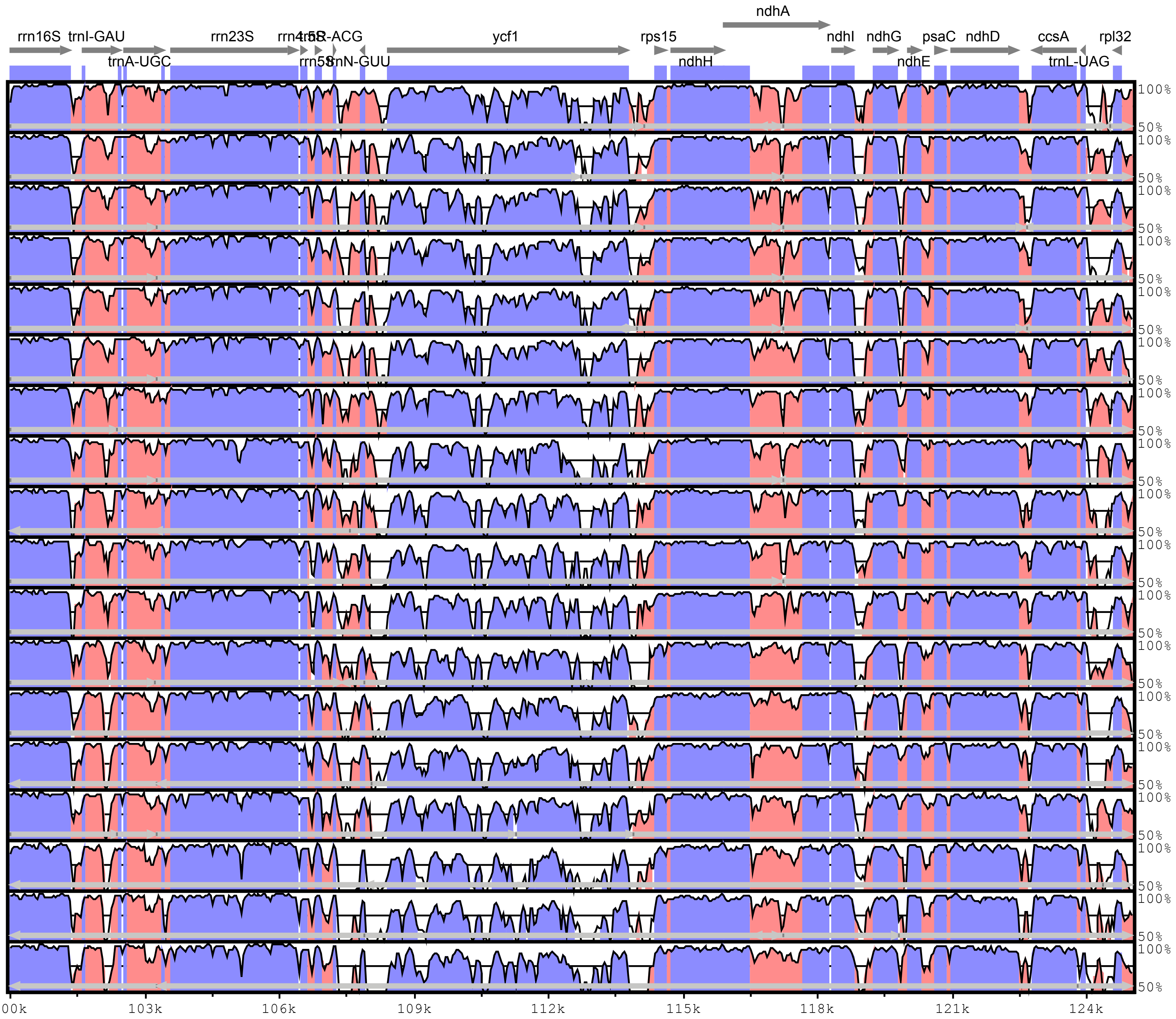
ndhA
rrn16S
trnI-GAU
rrn23S
rrn4.5S
trnR-ACG
ycf1
rps15
ndhI
ndhG
psaC
ndhD
ccsA
rpl32
trnA-UGC
rrn5S
trnN-GUU
ndhH
ndhE
trnL-UAG
100%
50%
100k
103k
106k
109k
112k
115k
118k
121k
124k

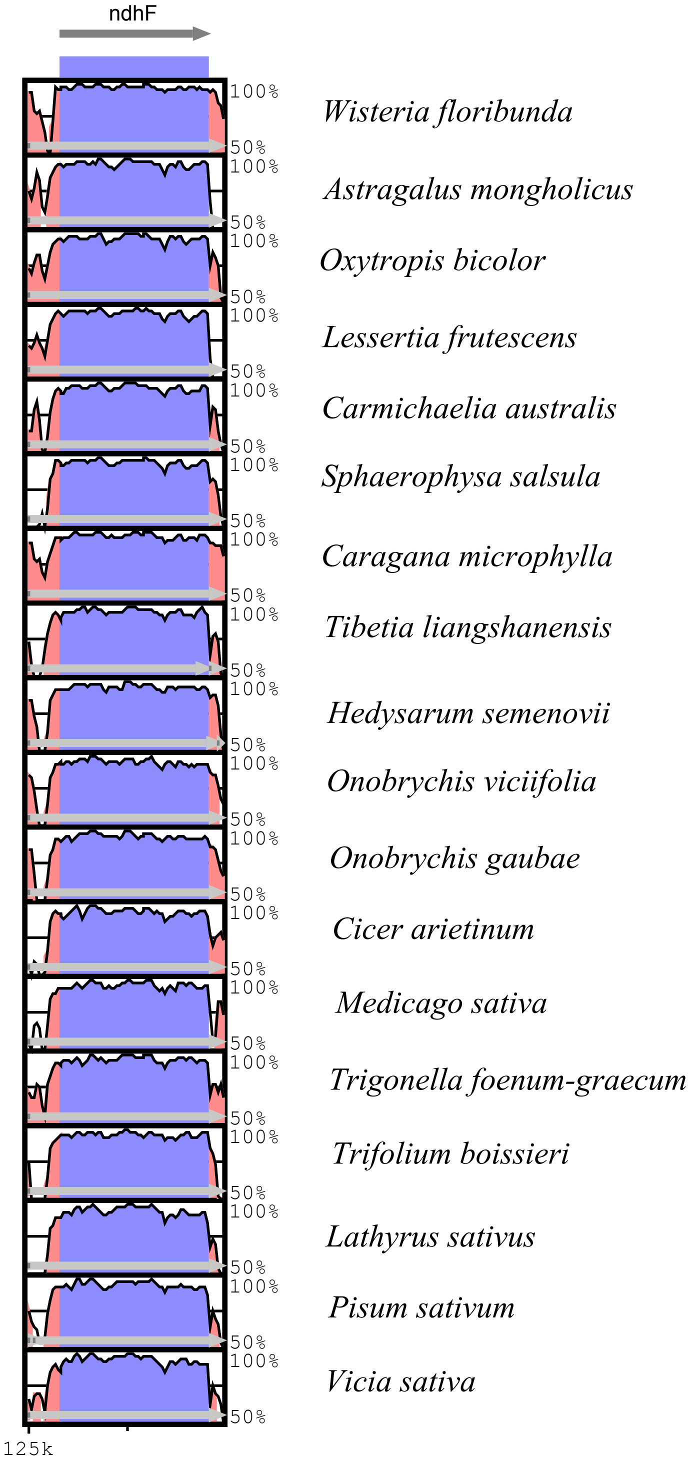

Pink bars represent noncoding sequences (CNS), and white peaks represent genomic differences. The y-axis represents the percentage identity (shown: 50-100%).
