# Supplementary material for: The complete chloroplast genome of Onobrychis gaubae (Fabaceae-Papilionoideae): comparative analysis with related IR-lacking clade species
Source: BMC Plant Biol. 2022 Feb 19;22:75. doi: 10.1186/s12870-022-03465-4 (PMC8858513; doi:10.1186/s12870-022-03465-4)
Supplement: Supplementary file 6 — Additional file 6: Table S9. The Ka, Ks and Ka/Ks ratio of IRLC chloroplast genome for individual genes and region. [file 12870_2022_3465_MOESM6_ESM.docx]

**Table S9.** The Ka, Ks and Ka/Ks ratio of IRLC chloroplast genome for individual genes and region.

| Gene groups | gene | Ka | Ks | Ka/Ks | Region |
| --- | --- | --- | --- | --- | --- |
| Large subunit of ribosomal proteins | *rpl33* | 0.021774 | 0.175307 | 0.124204 | LSC |
|  | *rpl20* | 0.094379 | 0.104231 | 0.905479 | LSC |
|  | *rpl36* | 0.025933 | 0.152872 | 0.169638 | LSC |
|  | *rpl14* | 0.02846 | 0.132922 | 0.21411 | LSC |
|  | *rpl16* | 0.030933 | 0.156628 | 0.197493 | LSC |
|  | *rpl2* | 0.025261 | 0.128452 | 0.196657 | IR |
|  | *rpl23* | 0.033557 | 0.091265 | 0.367687 | IR |
|  | *rpl32* | 0.06128 | 0.170782 | 0.35882 | SSC |
| Small subunit of ribosomal proteins | *rps2* | 0.030034 | 0.119335 | 0.251678 | LSC |
|  | *rps14* | 0.063579 | 0.175865 | 0.361521 | LSC |
|  | *rps4* | 0.020136 | 0.098541 | 0.204341 | LSC |
|  | *rps18* | 0.05959 | 0.134325 | 0.443625 | LSC |
|  | *rps11* | 0.043619 | 0.126872 | 0.343803 | LSC |
|  | *rps8* | 0.031763 | 0.18206 | 0.174464 | LSC |
|  | *rps3* | 0.059466 | 0.101634 | 0.585099 | LSC |
|  | *rps19* | 0.039163 | 0.088466 | 0.442689 | LSC |
|  | *rps7* | 0.043028 | 0.090718 | 0.474304 | IR |
|  | *rps15* | 0.055484 | 0.141945 | 0.390883 | SSC |
| DNA-dependent RNA polymerase | *rpoC2* | 0.027362 | 0.094618 | 0.289183 | LSC |
|  | *rpoC1* | 0.020766 | 0.109876 | 0.188994 | LSC |
|  | *rpoB* | 0.014184 | 0.109588 | 0.12943 | LSC |
|  | *rpoA* | 0.036295 | 0.096166 | 0.37742 | LSC |
| Photosystem I | *psaB* | 0.010148 | 0.101379 | 0.100099 | LSC |
|  | *psaA* | 0.007125 | 0.094294 | 0.075561 | LSC |
|  | *psaI* | 0.028605 | 0.108277 | 0.264183 | LSC |
|  | *psaJ* | 0.030623 | 0.127359 | 0.240446 | LSC |
|  | *psaC* | 0.01029 | 0.054383 | 0.189213 | SSC |
| Photosystem II | *psbA* | 0.000907 | 0.070853 | 0.012801 | LSC |
|  | *psbK* | 0.019547 | 0.110366 | 0.177110 | LSC |
|  | *psbI* | 0.002593 | 0.130447 | 0.019877 | LSC |
|  | *psbM* | 0.011653 | 0.087061 | 0.133848 | LSC |
|  | *psbD* | 0.00204 | 0.090585 | 0.02252 | LSC |
|  | *psbC* | 0.005462 | 0.10911 | 0.050059 | LSC |
|  | *psbZ* | 0.008697 | 0.097609 | 0.0891 | LSC |
|  | *psbJ* | 0.023621 | 0.078318 | 0.301603 | LSC |
|  | *psbL* | 0.000000 | 0.069069 | 0.000000 | LSC |
|  | *psbF* | 0.007306 | 0.038779 | 0.1884 | LSC |
|  | *psbE* | 0.001852 | 0.059055 | 0.03136 | LSC |
|  | *psbB* | 0.006695 | 0.10735 | 0.062366 | LSC |
|  | *psbT* | 0.024268 | 0.084139 | 0.288427 | LSC |
|  | *psbN* | 0.005574 | 0.072593 | 0.076784 | LSC |
|  | *psbH* | 0.019552 | 0.076608 | 0.255221 | LSC |
| NADH dehydrogenase | *ndhJ* | 0.012038 | 0.108054 | 0.111407 | LSC |
|  | *ndhK* | 0.01816 | 0.117726 | 0.154256 | LSC |
|  | *ndhC* | 0.015154 | 0.103892 | 0.145863 | LSC |
|  | *ndhB* | 0.01688 | 0.090195 | 0.18715 | IR |
|  | *ndhF* | 0.025373 | 0.110915 | 0.22876 | SSC |
|  | *ndhD* | 0.019733 | 0.102895 | 0.191778 | SSC |
|  | *ndhE* | 0.008238 | 0.10369 | 0.079448 | SSC |
|  | *ndhG* | 0.018175 | 0.103404 | 0.175766 | SSC |
|  | *ndhI* | 0.006527 | 0.09083 | 0.071859 | SSC |
|  | *ndhA* | 0.013316 | 0.117003 | 0.113809 | SSC |
|  | *ndhH* | 0.009921 | 0.10536 | 0.094162 | SSC |
| Cytochrome b/f complex | *petN* | 0.003949 | 0.045575 | 0.086648 | LSC |
|  | *petA* | 0.017335 | 0.132417 | 0.130912 | LSC |
|  | *petL* | 0.028662 | 0.111875 | 0.256196 | LSC |
|  | *petG* | 0.01103 | 0.149788 | 0.073637 | LSC |
|  | *petB* | 0.002989 | 0.117049 | 0.025536 | LSC |
|  | *petD* | 0.003415 | 0.133447 | 0.02559 | LSC |
| ATP synthase | *atpA* | 0.01674 | 0.114011 | 0.146827 | LSC |
|  | *atpF* | 0.04361 | 0.130942 | 0.333506 | LSC |
|  | *atpH* | 0.007309 | 0.121581 | 0.060116 | LSC |
|  | *atpI* | 0.009415 | 0.108375 | 0.086874 | LSC |
|  | *atpE* | 0.044808 | 0.093971 | 0.476827 | LSC |
|  | *atpB* | 0.016409 | 0.125962 | 0.130269 | LSC |
| RubisCo large subunit | *rbcL* | 0.016842 | 0.144967 | 0.116178 | LSC |
| Maturase K | *matK* | 0.039602 | 0.097343 | 0.406829 | LSC |
| Envelope membrane protein | *cemA* | 0.058693 | 0.136592 | 0.429695 | LSC |
| Subunit of acetyl-CoAcarboxylase | *accD* | 0.102693 | 0.044552 | 2.305014 | LSC |
| C-type cytochrome synthesis gene | *ccsA* | 0.020907 | 0.114176 | 0.183112 | SSC |
| Protease | *clpP* | 0.146771 | 0.232273 | 0.63189 | LSC |
| Conserved hypothetical chloroplast open reading frames | *ycf3* | 0.013951 | 0.107846 | 0.12936 | LSC |
|  | *ycf4* | 0.165691 | 0.169432 | 0.97792 | LSC |
|  | *ycf2* | 0.097794 | 0.150866 | 0.648217 | IR |
|  | *ycf1* | 0.147039 | 0.181067 | 0.812069 | SSC |

*Ka*: non-synonymous substitution, *Ks*: synonymous substitution, LSC: Large Single Copy, SSC: Small Single Copy, IR: Inverted Repeat.
