# Supplementary material for: The complete chloroplast genome of Onobrychis gaubae (Fabaceae-Papilionoideae): comparative analysis with related IR-lacking clade species
Source: BMC Plant Biol. 2022 Feb 19;22:75. doi: 10.1186/s12870-022-03465-4 (PMC8858513; doi:10.1186/s12870-022-03465-4)
Supplement: Supplementary file 7 — Additional file 7: Table S10. Prediction of RNA editing sites in chloroplast genes of O. gaubae. Table S11. Prediction of RNA editing sites in chloroplast genes of O. viciifolia. [file 12870_2022_3465_MOESM7_ESM.docx]

**Table S10**. Prediction of RNA editing sites in chloroplast genes of *O*. *gaubae*.

| Gene | Nucleotide position | Amino acid position | RNA editing effect | Score |
| --- | --- | --- | --- | --- |
| *acc*D | 275  346  1144 | 92  116  382 | CCG (P) => CTG (L)  CAT (H) => TAT (Y)  CCG (P) => TCG (S) | 1.00  1.00  1.00 |
| *atp*F | 1093 | 365 | CTT (L) => TTT (F) | 0.86 |
| *ccs*A | 137  514 | 46  172 | ACA (T) => ATA (I)  CTT (L) => TTT (F) | 1.00  0.86 |
| *mat*K | 985  1184 | 329  395 | CTT (L) => TTT (F)  TCA (S) => TTA (L) | 0.86  0.86 |
| *ndh*A | 137  341  623  1156  1168  1355 | 46  114  208  386  390  452 | GCA (A) => GTA (V)  TCA (S) => TTA (L)  ACT (T) => ATT (I)  CTC (L) => TTC (F)  CCG (P) => TCG (S)  CCA (P) => CTA (L) | 1.00  1.00  1.00  1.00  1.00  1.00 |
| *ndh*B | 95  413  532  557  692  878  1340  1427  2012 | 32  138  178  186  231  293  447  476  671 | TCA (S) => TTA (L)  TCA (S) => TTA (L)  CAT (H) => TAT (Y)  TCA (S) => TTA (L)  TCT (S) => TTT (F)  TCA (S) => TTA (L)  CCG (P) => CTG (L)  TCG (S) => TTG (L)  ACC (T) => ATC (I) | 1.00  1.00  1.00  0.80  1.00  1.00  1.00  1.00  1.00 |
| *ndh*D | 5  677  881  1408 | 2  226  294  470 | ACG (T) => ATG (M)  TCG (S) => TTG (L)  TCA (S) => TTA (L)  CTT (L) => TTT (F) | 1.00  1.00  1.00  0.80 |
| *ndh*F | 13  241  290  1172 | 5  81  97  391 | CAT (H) => TAT (Y)  CTT (L) => TTT (F)  TCG (S) => TTG (L)  GCG (A) => GTG (V) | 1.00  1.00  1.00  0.80 |
| *ndh*G | 166  314  385 | 56  105  129 | CAT (H) => TAT (Y)  ACA (T) => ATA (I)  CCA (P) => TCA (S) | 0.80  0.80  0.80 |
| *pet*B | 821  907  973  1174  1210  1213  1357 | 274  303  325  392  404  405  453 | CCT (P) => CTT (L)  CTC (L) => TTC (F)  CGG (R) => TGG (W)  CTT (L) => TTT (F)  CAT (H) => TAT (Y)  CCT (P) => TCT (S)  CCA (P) => TCA (S) | 1.00  1.00  1.00  1.00  1.00  1.00  1.00 |
| *pet*D | 1048  1108  1175 | 350  370  392 | CTT (L) => TTT (F)  CTT (L) => TTT (F)  CCT (P) => CTT (L) | 1.00  1.00  1.00 |
| *psa*B | 1456 | 486 | CTT (L) => TTT (F) | 1.00 |
| *psb*L | 106 | 36 | CTT (L) => TTT (F) | 1.00 |
| *rpl*2 | 262 | 88 | CAC (H) => TAC (Y) | 1.00 |
| *rpo*B | 338  551  566  718  2000  2426 | 113  184  189  240  667  809 | TCT (S) => TTT (F)  TCA (S) => TTA (L)  TCG (S) => TTG (L)  CCT (P) => TCT (S)  TCT (S) => TTT (F)  TCA (S) => TTA (L) | 1.00  1.00  1.00  1.00  1.00  0.86 |
| *rpo*C1 | 127  2306 | 43  769 | CTT (L) => TTT (F)  ACG (T) => ATG (M) | 0.86  0.86 |
| *rpo*C2 | 3725 | 1242 | TCA (S) => TTA (L) | 0.86 |
| *rps*2 | 248 | 83 | TCA (S) => TTA (L) | 1.00 |
| *rps*14 | 101 | 34 | GCT (A) => GTT (V) | 0.86 |

**Table S11**. Prediction of RNA editing sites in chloroplast genes of *O*. *viciifolia*.

| Gene | Nucleotide position | Amino acid position | RNA editing effect | Score |
| --- | --- | --- | --- | --- |
| *acc*D | 163  290  1162  1510 | 55  97  388  504 | CCG (P) => TCG(S) CCA (P) => CTA (L)  CCT (P) => TCT (S)  CCG (P) => TCG (S) | 1.00  1.00  1.00  1.00 |
| *atp*I | 157 | 53 | CCA (P) => TCA (S) | 1.00 |
| *ccs*A | 137  514 | 46  172 | ACA (T) => ATA (I)  CTT (L) => TTT (F) | 1.00  0.86 |
| *clp*P | 355  416 | 119  139 | CTC (L) => TTC (F)  TCT (S) => TTT (F) | 0.86  1.00 |
| *mat*K | 979  1178 | 327  393 | CTT (L) => TTT (F)  TCA (S) => TTA (L) | 0.86  0.86 |
| *ndh*A | 137  341  1961 | 46  114  654 | GCA (A) => GTA (V)  TCA (S) => TTA (L)  TCA (S) => TTA (L) | 1.00  1.00  1.00 |
| *ndh*B | 95  413  557  692  758  1187  1207 | 32  138  186  231  253  396  403 | TCA (S) => TTA (L)  TCA (S) => TTA (L)  TCA (S) => TTA (L)  TCT (S) => TTT (F)  TCT (S) => TTT (F)  ACA (T) => ATA(I)  CTC (L) => TTC (F) | 1.00  1.00  0.80  1.00  1.00  1.00  1.00 |
| *ndh*D | 5  677  881  1408 | 2  226  294  470 | ACG (T) => ATG (M)  TCG (S) => TTG (L)  TCA (S) => TTA (L)  CTT (L) => TTT (F) | 1.00  1.00  1.00  0.80 |
| *ndh*F | 13  241  290  1172 | 5  81  97  391 | CAT (H) => TAT (Y)  CTT (L) => TTT (F)  TCG (S) => TTG (L)  GCG (A) => GTG (V) | 1.00  1.00  1.00  0.80 |
| *ndh*G | 166  314  385 | 56  105  129 | CAT (H) => TAT (Y)  ACA (T) => ATA (I)  CCA (P) => TCA (S) | 0.80  0.80  0.80 |
| *pet*B | 766  862  928  1129  1165  1168  1315  1354  1373 | 256  288  310  377  389  390  439  452  458 | CTT (L) => TTT (F) CTC (L) => TTC (F)  CGG (R) => TGG (W)  CTT (L) => TTT (F)  CAT (H) => TAT (Y)  CCT (P) => TCT (S)  CCT (P) => TCT (S)  CTC (L) => TTC (F)  TCA (S) => TTA (L) | 1.00  1.00  1.00  1.00  1.00  1.00  1.00  1.00  1.00 |
| *pet*D | 38  601 | 13  201 | GCA (A) => GTA (V) CCT (P) => TCT (S) | 1.00  1.00 |
| *psa*B | 1456 | 486 | CTC (L) => TTC (F) | 1.00 |
| *psb*F | 77 | 26 | TCT (S) => TTT (F) | 1.00 |
| *psb*L | 2 | 1 | ACG (T) => ATG (M) | 1.00 |
| *rpl*2 | 262 | 88 | CAC (H) => TAC (Y) | 1.00 |
| *rpo*B | 338  551  566  718  2000  2426 | 113  184  189  240  667  809 | TCT (S) => TTT (F)  TCA (S) => TTA (L)  TCG (S) => TTG (L)  CCT (P) => TCT (S)  TCT (S) => TTT (F)  TCA (S) => TTA (L) | 1.00  1.00  1.00  1.00  1.00  0.86 |
| *rpo*C1 | 41  127  577  731  1325  1435  1784  2287 | 14  43  193  244  442  479  595  763 | TCA (S) => TTA (L) CTT (L) => TTT (F)  CCA (P) => TCA (S)  TCT (S) => TTT (F)  GCG (A) => GTG (V)  CCG (P) => TCG (S)  GCG (A) => GTG (V)  CTC (L) => TTC (F) | 1.00  0.86  1.00  0.86  1.00  1.00  1.00  1.00 |
| *rpo*C2 | 3752 | 1251 | TCA (S) => TTA (L) | 0.86 |
| *rps*2 | 248 | 83 | TCA (S) => TTA (L) | 1.00 |
| *rps*14 | 101 | 34 | GCT (A) => GTT (V) | 0.86 |
| *ycf*3 | 1471 | 491 | CCC (P) => TCC (S) | 0.86 |
