# Supplementary material for: The complete chloroplast genome of Onobrychis gaubae (Fabaceae-Papilionoideae): comparative analysis with related IR-lacking clade species
Source: BMC Plant Biol. 2022 Feb 19;22:75. doi: 10.1186/s12870-022-03465-4 (PMC8858513; doi:10.1186/s12870-022-03465-4)
Supplement: Supplementary file 8 — Additional file 8: Table S12. Accession number and sampled chloroplast genomes obtained from GenBank. [file 12870_2022_3465_MOESM8_ESM.docx]

**Table S12**. Accession number and sampled chloroplast genomes obtained from GenBank.

| **Species** | **Accession** | **Genome Size** |
| --- | --- | --- |
| **IRLC** | | |
| *Alhagi sparsifolia* | MT571455 | 123,233 bp |
| *Astragalus mongholicus* | KU666554 | 123,582 bp |
| *Astragalus bhotanensis* | MN709865 | 123,278 bp |
| *Callerya nitida* | MT120748 | 132,319 bp |
| *Caragana korshinskii* | KX289923 | 129,331 bp |
| *Caragana microphylla* | KX289922 | 130,029 bp |
| *Carmichaelia australis* | MF597719 | 122,805 bp |
| *Cicer arietinum* | EU835853 | 125,319 bp |
| *Glycyrrhiza glabra* | KF201590 | 127,943 bp |
| *Glycyrrhiza triphylla* | MT120806 | 127,735 bp |
| *Hedysarum semenovii* | MN709827 | 123,407 bp |
| *Hedysarum taipeicum* | MK426698 | 126,699 bp |
| *Lathyrus sativus* | HM029371 | 121,020 bp |
| *Lens culinaris* | KF186232 | 122,967 bp |
| *Lessertia frutescens* | MF286764 | 122,700 bp |
| *Medicago sativa* | MK460489 | 125,330 bp |
| *Melilotus albus* | MH191352 | 127,205 bp |
| *Onobrychis viciifolia* | MW007721 | 121,932 bp |
| *Oxytropis bicolor* | MN255323 | 122,461 bp |
| *Oxytropis splendens* | MT409174 | 122,318 bp |
| *Pisum sativum* | HM029370 | 122,169 bp |
| *Sphaerophysa salsula* | MW122834 | 123,300 bp |
| *Tibetia liangshanensis* | MF193597 | 123,372 bp |
| *Trifolium boissieri* | KJ788284 | 125,740 bp |
| *Trigonella foenum-graecum* | MK460508 | 125,645 bp |
| *Vicia sativa* | KJ850242 | 122,467 bp |
| *Wisteria floribunda* | MT120817 | 130,561 bp |
|  | **Outgroup** |  |
| *Lotus japonicus* | AP002983 | 150,519 bp |
| *Robinia pseudoacacia* | KJ468102 | 154,835 bp |
